# Wireless Electromagnetic Generation of miRNA Sponges and Nerve Stimulation by an Adaptable Electrical Scaffold for Repair of Traumatic Brain Injury

**DOI:** 10.1021/acsnano.6c04759

**Published:** 2026-05-25

**Authors:** Hoi Man Iao, Wan-Chi Pan, Yun-Hsuan Chang, Ngoc-Tri Tran, Hsiu-Ching Liu, Ru-Siou Hsu, Tsu-Chin Chou, I-Chi Lee, Lun-De Liao, Wen-Hsuan Chiang, Ssu-Ju Li, Eric Hwang, Ming-You Shie, You-Yin Chen, Shang-Hsiu Hu

**Affiliations:** † Department of Biomedical Engineering and Environmental Sciences, 34881 National Tsing Hua University, Hsinchu 300044, Taiwan; ‡ Department of Biomedical Engineering, 390197 National Yang Ming Chiao Tung University, Taipei 112304, Taiwan; § Institute of Analytical and Environmental Sciences, National Tsing Hua University, Hsinchu 300044, Taiwan; ∥ Institute of Biomedical Engineering and Nanomedicine, National Health Research Institutes, Miaoli County 35053, Taiwan; ⊥ Department of Chemical Engineering, National Chung Hsing University, Taichung 402, Taiwan; # Institute of Molecular Medicine and Bioengineering, National Yang Ming Chiao Tung University, Hsinchu 300093, Taiwan; ¶ Department of Biological Science and Technology, National Yang Ming Chiao Tung University, Hsinchu 300093, Taiwan; ∇ Center for Intelligent Drug Systems and Smart Bio-devices (IDS2B), National Yang Ming Chiao Tung University, Hsinchu 300093, Taiwan; ○ Institute of Biomedical Engineering, National Tsing Hua University, Hsinchu 300044 Taiwan; ⧫ Department of Biomedical Engineering, 38020 China Medical University, Taichung 406040, Taiwan; †† Department of Bioinformatics and Medical Engineering, Asia University, Taichung 41354, Taiwan; ‡‡ Xenotransplantation Translational Research Center, China Medical University Hospital, Taichung 404332, Taiwan

**Keywords:** conductive polymer composites, wireless bioelectrionics, in vivo transfection, electrical stimulus, nerve regeneration

## Abstract

Bioelectronic transduction and microRNA (miRNA) regulation play crucial roles in shaping neuronal cell fate and supporting brain repair. However, clinical progress remains limited due to the lack of tools to perform spatiotemporal regulation of neuronal electrical activity and nonviral gene regulation in vivo. In this study, an adaptable electronic scaffold (AES) that functions both as an antenna and gene transfection agent was developed for neuron miRNA modulation in traumatic brain injury (TBI). The intrinsic properties of AES alleviate inflammation and glial scarring after TBI by suppressing activated microglia and stellate cells. Under high-frequency magnetic field (HFMF) stimulation, the “wireless messenger” generates localized electrical cues that aid in restoring brain function as well as enhancing neuronal uptake of gene therapeutics via electroporation. In neurons, AES-induced Eddy currents and mechanical forces further promote endosome escape and the formation of miRNA sponges both in vitro and in vivo, thereby reducing the increase in miR-6236 levels after neuronal injury. The synergic effects achieve in situ gene modulation, promotes neurite outgrowth, and enhance angiogenesis within the lesion. In whole-brain diffusion-weighted magnetic resonance imaging (dMRI), the signal bundles reveal improved intercortical and cortical stellate fiber connectivity, and a recovery in motor function. In summary, this wirelessly driven gene regulation platform combines miRNA-targeted therapy with bioelectronic stimulation to achieve precise neuroregenerative intervention.

## Graphical abstract

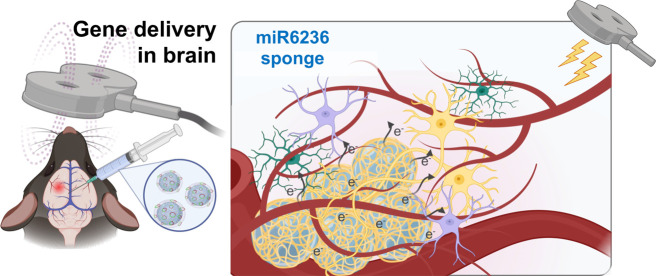

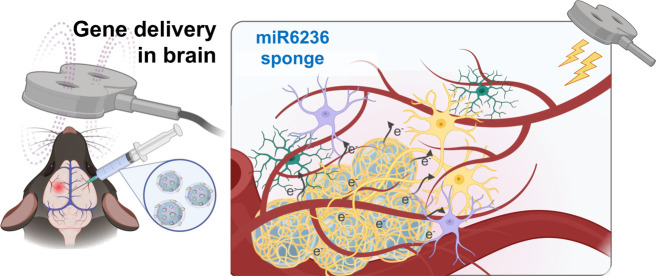

## 1 Introduction

Traumatic brain injury (TBI) is a leading cause of long-term disability, marked by inflammation, glial activation, and limited endogenous repair.^1,2^ Although adult neural stem cells (NSCs) persist in the subventricular zone and hippocampus, their regenerative capacity in the injured cortex is minimal.^3^ Gene modulation has shown promise for neuronal repair, as dysregulated miRNA expression can further impair recovery.^4,5^ For example, the upregulation of miR-21 and miR-155 contributes to apoptotic and inflammatory signaling, exacerbating neurotoxicity.^6,7^ miR-181a is known to sensitize neurons to excitotoxicity and mitochondrial stress, while miR-34a promotes neuronal apoptosis and impairs synaptic plasticity.^8,9^ Injury-associated miRNAs promote microglial invasion and stellate cell activation, intensifying inflammation and glial scarring.^10,11^ These processes drive cortical atrophy, which in turn restricts neuronal regeneration and perpetuates a cycle of progressive neuron loss.^12,13^ Despite advances in neuronal engineering, effective regeneration remains challenging due to cellular heterogeneity, inefficient gene modulation, and the complex pathology of TBI.

To address these issues on neural repair, strategies have focused on engineering the damaged tissue environment and directly modulating gene expression.^14,15^ Microporous annealed particulate scaffolds (MAPS), a type of granular scaffold, preserve structural integrity while supporting nerve regeneration.^16,17^ Their interconnected pores reduce scarring, enhance neuronal infiltration, and modulate immune responses by balancing macrophage activation.^18,19^ Complementing this approach, targeted delivery of therapeutic nucleic acids allows precise regulation of miRNA and gene expression, offering high specificity for neurodegenerative disease treatment.^20,21^ Preclinical studies using AAV-based CRISPR therapies aim to edit genes responsible for toxic protein accumulation, such as alpha-synuclein in Parkinson’s and Alzheimer’s disease.^22,23^ However, safety concerns with viral vectors underscore the need for engineered nonviral delivery systems suitable for brain applications.

In damaged neurons, miR-6236 functions as a potent negative regulator that disrupts the transcriptomic and cytoskeletal networks required for neurite outgrowth, axonal elongation, and structural maturation.^24^ This prior work demonstrated that its pathological upregulation under adverse conditions creates an intrinsic barrier to neural repair by severely impairing microtubule organization, significantly hindering the regenerative capacity of both central and peripheral neurons. Notably, miR-6236 has also been identified in an independent brain-derived extracellular vesicle miRNA profiling study as part of an injury-responsive biomarker panel distinguishing TBI from sham controls, further supporting its pathological relevance in the TBI context.^25^ Although miRNA sponges delivered by viral vectors can deplete miR-6236 in vitro, in vivo neuronal gene delivery remains hindered by the restrictive neuronal membrane and low endocytic activity.^26^ Recent advances have introduced mRNA-loaded lipid nanoparticles (LNPs) optimized through AI-based validation, in vitro central nervous system (CNS) models and mouse studies, enabling acetylcholine-coupled LNPs to target the brain via AchR-mediated uptake and preferential neuronal transfection.^27^ Neurotransmitter-derived LNPs have also been developed to cross the blood–brain barrier (BBB) and deliver diverse cargoes, including antisense oligonucleotides and gene-editing proteins, following intravenous administration.^28,29^ In addition, protoberberine-inspired ionizable lipids that exploit dopamine D3 receptor-mediated endocytosis enhance BBB penetration and support mRNA delivery in murine models of Alzheimer’s disease.^30,31^ Yet, despite these advances, efficient in vivo gene delivery for neuronal repair remains constrained by the complexity and sensitivity of the neural microenvironment.^32^

Electrical stimulation has been recognized as a potent modulator of neural repair, facilitating to drive neurite extension, bolster synaptic plasticity and temper glial activation.^33^ Although implanted electrodes provide compelling proof-of-concept for the regenerative value, their limited spatial fidelity and mechanical mismatch with soft neural tissue constrain their translational potential.^34,35^ Conductive hydrogels have emerged as soft, tissue-mimetic matrices that couple mechanical compliance with the ability to relay electrical signals in the brain.^36,37^ These materials can bridge lesion cavities, support cellular infiltration and form stable long-term bioelectronic interfaces, and have been applied across cardiac, peripheral and central nerve repair.^38–40^ Yet most conductive hydrogels provide only passive electrical support and lack the molecular specificity needed to modulate gene-regulatory programmes essential for regeneration. This gap underscores the need for strategies that integrate the precision of miRNA-based modulation with the restorative capacity of electrically active hydrogels. To achieve noninvasive electrical modulation deep within the brain, the choice of external biophysical stimuli is critical. Unlike near-infrared light, which is heavily scattered by bone and dense tissue, or ultrasound, where neurochemical mechanisms and functional connectivity effects remain complex and poorly understood,^41^ magnetic fields offer deep tissue penetration and minimal attenuation in biological tissue. High-frequency magnetic fields (HFMF) can penetrate the intact cranium and deep brain tissue with minimal attenuation, serving as a robust energy source for wireless, nanomaterial-mediated neuromodulation.^42^

By leveraging this electric capability, a wirelessly bioelectronic platform that unites miRNA-targeted regulation with remote control of neuronal activity to overcome long-standing barriers in neuro-regenerative therapy was developed. This adaptable electronic scaffold (AES) composed of conductive poly­(3,4-ethylenedioxythiophene)/poly­(styrenesulfonate) (PEDOT:PSS) and ultrasmall Au-decorated carbon dots (AuCDs) integrates antenna-like electromagnetic responsiveness with gene-delivery capability, enabling spatiotemporally precise modulation of neuronal miRNA programs in vivo (Figure 1). Leveraging its intrinsic anti-inflammatory and antiscarring properties, AES mitigates microglial and astroglial activation after traumatic brain injury, while high-frequency magnetic stimulation drives localized electrical cues that enhance neuronal uptake of gene therapeutics and facilitate endosomal escape. By promoting the formation of miRNA sponges and counteracting injury-induced upregulation of miR-6236, AES achieves in situ molecular reprogramming that supports neurite outgrowth, angiogenesis and circuit-level restoration. Whole-brain diffusion MRI further reveals improved intercortical and stellate fiber connectivity, accompanied by functional recovery. Together, these findings establish a wirelessly driven, nonviral gene-modulation strategy that couples bioelectronic transduction with miRNA precision to advance next-generation neuroregenerative interventions.

**Figure 1:**
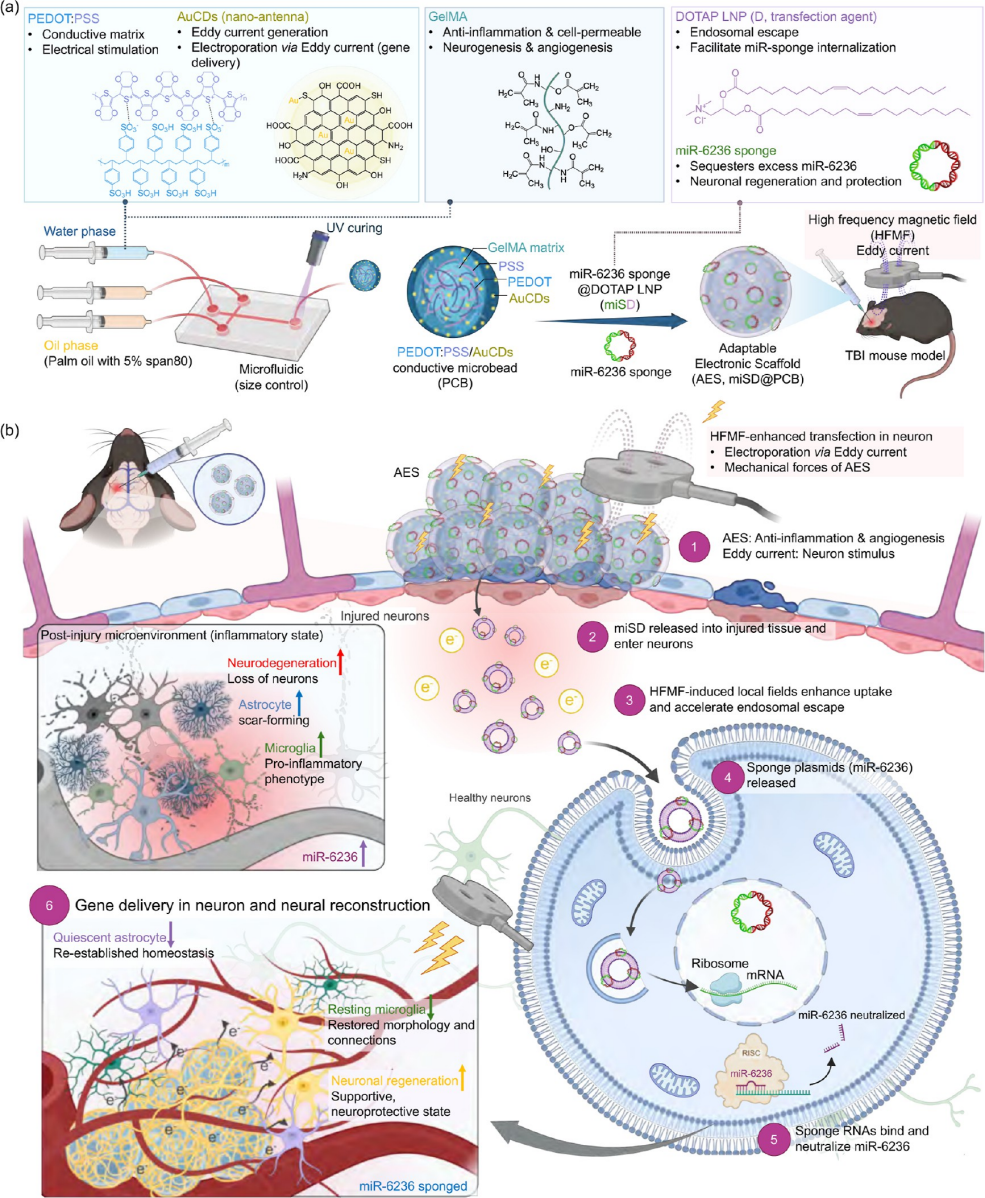
HFMF-responsive AES for gene transfection and neural repair in TBI. Upon a HFMF stimulation, the AES, wireless nanomessenger, generates localized electrical cues that help restore neural activity and enhance intracellular delivery of gene therapeutics via electroporation. In neurons, AES-induced eddy currents and magneto-mechanical forces facilitate endosomal escape and promote formation of miRNA-binding “sponges,” thereby preventing the injury-induced rise of miR-6236 both in vitro and in vivo.

## 2 Results and Discussion

### 2.1 Synthesis and Characterization of Au-Decorated Carbon Dots (Nanoantennas)

This versatile AES was prepared using AuCDs-assisted approach, employing a conductive microbead comprising nucleic acids (Figure 1a). AuCDs as nanoantennas were synthesized via a one-pot aqueous route using glutathione (GSH) and HAuCl_4_ under mild heating (Figure 2a). Transmission electron microscopy (TEM) revealed ultrasmall, uniformly dispersed nanodots (average 2.6 ± 0.3 nm) with lattice fringes of 0.2–0.24 nm and no evidence of aggregation (Figure 2b). Elemental mapping by TEM–EDS confirmed the spatial coexistence of Au, C, N, O, and S (Figure S1). Particle-size analysis from multiple fields indicated a narrow distribution with an average diameter of 2.64 ± 0.27 nm (Figure 2c). X-ray photoelectron spectroscopy (XPS) detected C, N, O, S, and Au at atomic percentages of 54.5%, 28.5%, 10.0%, 3.6%, and 3.4%, respectively (Figures 2d–h and S2). The high-resolution C 1s and N 1s spectra showed carbonyl- and amine-related components characteristic of GSH-derived domains, while the Au 4f_7_/_2_ peak at 84.8 eV exhibited a slight positive shift relative to metallic Au^0^ (84.0 eV). Correspondingly, the S 2p_3_/_2_ component at 163.3 eV is consistent with thiolate-type S bound to Au and/or C, confirming partial Au–S coordination within the carbon framework. Fourier-transform infrared (FTIR) spectra showed characteristic O–H/N–H (∼3270 cm^–1^), C=O (∼1720 cm^–1^), and C–N (∼1530 cm^–1^) vibrations of GSH-derived surface groups, while the disappearance of the S–H band (∼2520–2600 cm^–1^) indicated thiol consumption during Au coordination (Figure 2i), further indicating thiol involvement in the reaction.

**Figure 2:**
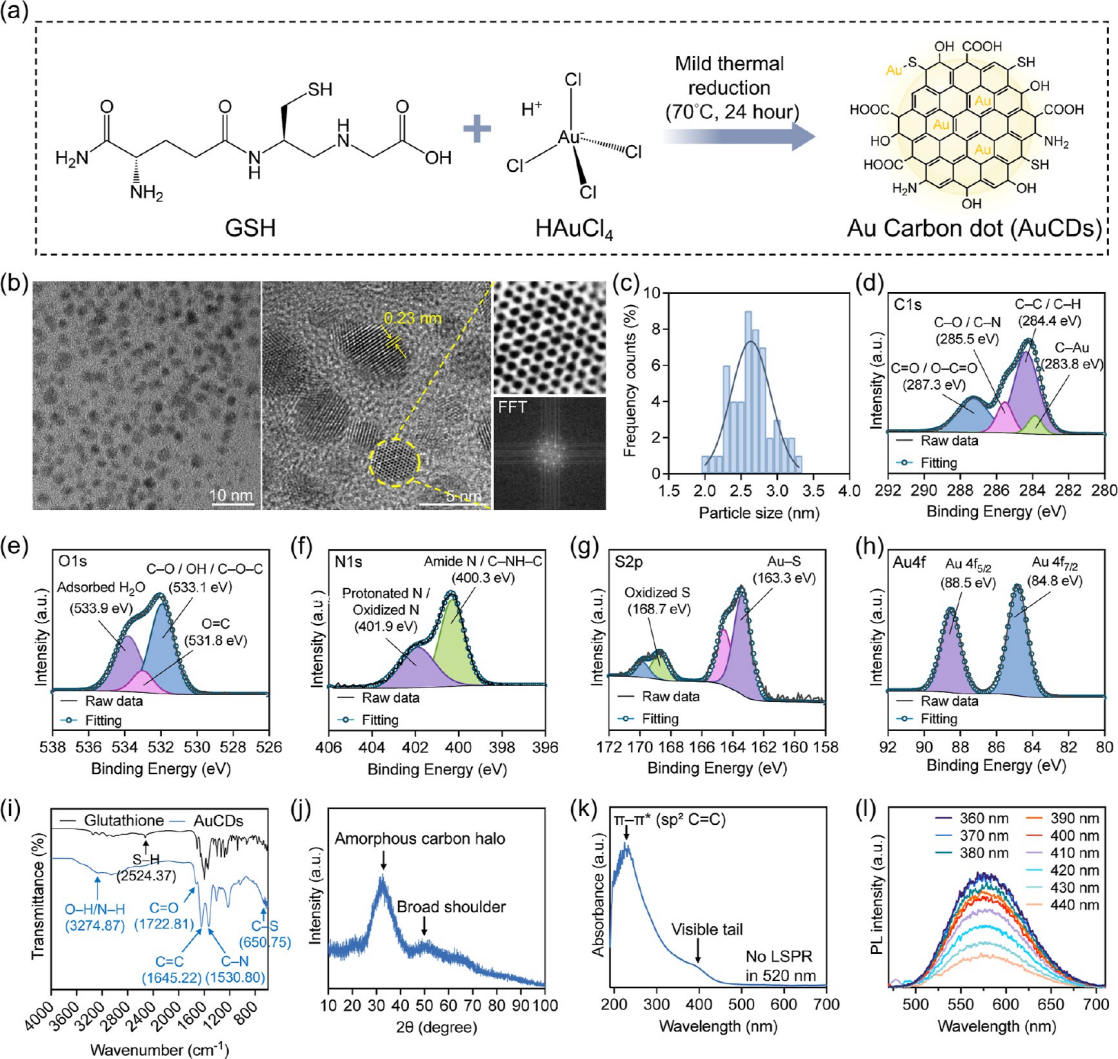
Synthesis and physicochemical characterization of gold–carbon nanocomposites. (a) Schematic representation of the AuCD synthesis route from l-glutathione (GSH) and chloroauric acid (HAuCl_4_) precursors. (b) TEM and high-resolution TEM images reveal ultrasmall, uniform nanoparticles with lattice fringes of approximately 0.25 nm. (c) Particle size distribution fitted with a Gaussian model. (d–h) High-resolution X-ray photoelectron spectra identify C 1s, N 1s, O 1s, S 2p and Au 4f peaks corresponding to well-defined oxidation states. (i) Fourier-transform infrared spectra verify the presence of hydrophilic functional group. (j) X-ray diffraction pattern, vertical dashed lines mark fcc Au reflections (111, 200, 220, 311). (k) Ultraviolet–visible absorption spectrum. (l) Photoluminescence spectra under 360–440 nm excitation shows a broad.

X-ray diffraction (XRD) displayed a broad amorphous halo centered at ∼32° (2θ) with a weak, similarly broad shoulder near ∼49°, characteristic of short-range ordered carbonaceous domains. Notably, no sharp reflections corresponding to gold were detected at ∼38.2°, 44.4°, 64.6°, or 77.5°, consistent with the absence of discrete Au nanoparticles and the formation of ultrasmall Au-decorated carbon dots (Figure 2j).^43^ Ultraviolet–visible spectra exhibited a strong absorption band at ∼225 nm assignable to the π–π* transition of sp^2^ carbon, accompanied by a monotonic tail extending into the visible region and no discernible plasmon resonance near 520 nm (Figure 2k). Photoluminescence measurements revealed a broad, nearly excitation-independent emission centered at ∼575 nm across 360–440 nm excitation, characteristic of surface/molecular-state-dominated carbon dots rather than metal nanoclusters (Figures 2l and S3).^44^ These data confirm the nanoscale dispersion and hybrid composition suitable for use as a conductive material within the microbead system. Beyond their electrical function, exploratory TEMP–EPR and TMB assays (Figure S4) revealed attenuated ^1^O_2_ and peroxidase-like activity, suggesting redox-interactive surface states.^45^

### 2.2 Synthesis and Characterization of PEDOT:PSS/AuCDs Conductive Microbeads

The core of AES was prepared using a microfluidic chip to generate monodisperse conductive microbeads (MB). At the T-shaped microfluidic junction, the water phase containing PEDOT:PSS (poly­(3,4-ethylenedioxythiophene) polystyrenesulfonate), GelMA (gelatin methacryloyl) and AuCDs formed a laminar aqueous flow that was sheared by the oil phase to yield a stable water-in-oil emulsion. The resulting droplets passed through the capillary channel and were photo-cross-linked under UV irradiation (365 nm, OmniCure 1500 series), producing uniform PEDOT:PSS/AuCDs conducting microgels (PCB) (Figure 3a). The obtained PCB exhibited highly uniform size, spherical morphology, and smooth surfaces (Figure 3b). Element mapping further confirmed the incorporation of PEDOT:PSS and AuCDs within PCB, as evidenced by the clear detection of Au and S signals in PCB (Figures 3c and S5).

**Figure 3:**
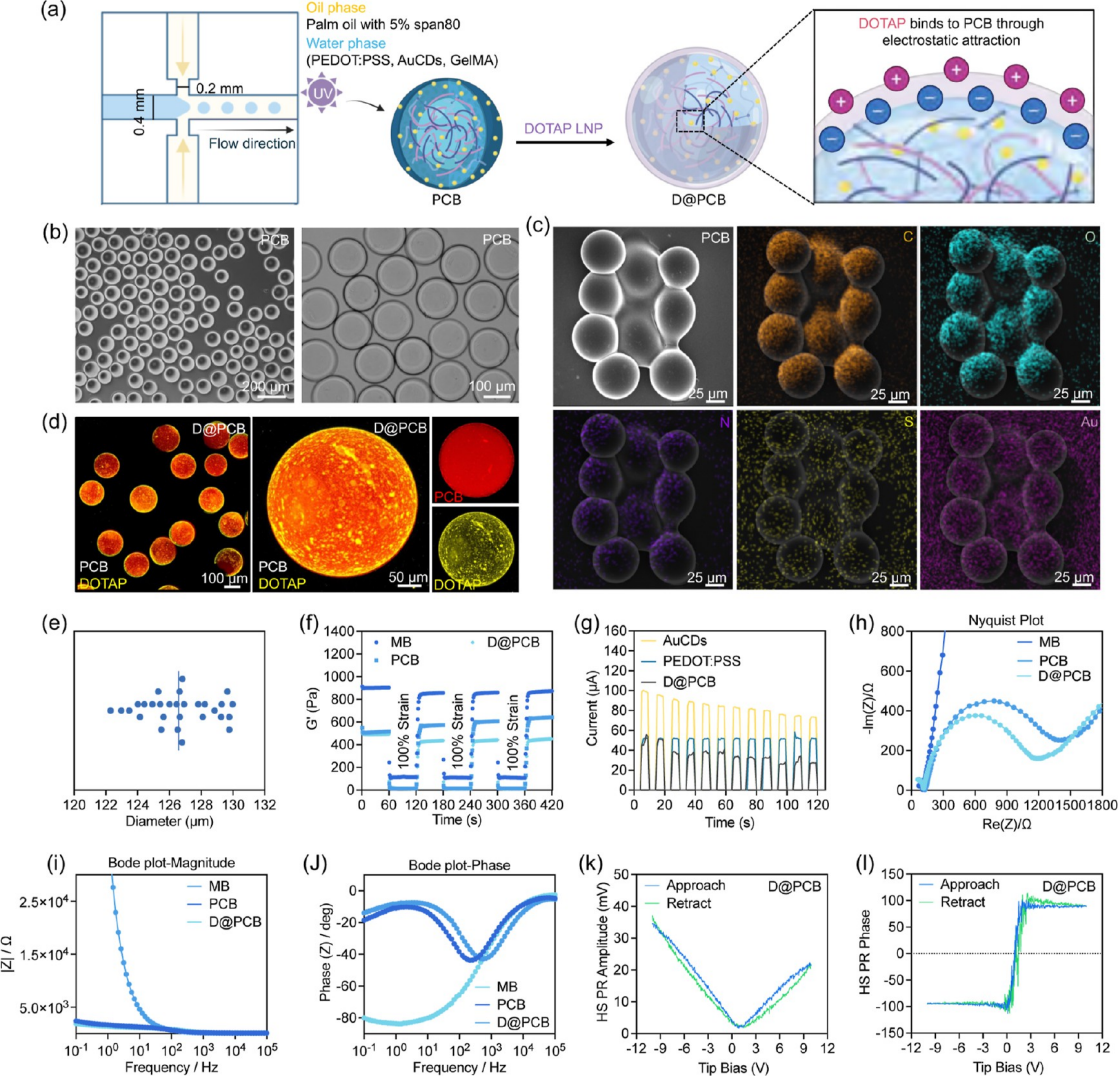
Fabrication and multifunctional characterization of conductive microbeads (D@PCB). (a) Microfluidic generation of monodisperse PCB followed by DOTAP coating to form D@PCB. (b) Bright-field microscopy reveals uniform particle size and dispersion. (c) Scanning electron microscopy and elemental mapping confirm smooth morphology. (d) CLMS imaging shows DOTAP coating (yellow) surrounding the PCB core (red), verifying surface modification. (e) Size-distribution analysis demonstrates stable water-in-oil droplet templating during emulsification. (f) Step-strain rheology indicates rapid self-healing and recovery of viscoelastic networks. (g) Current–time (*I*–*T*) responses display dynamic current modulation under intermittent HFMF stimulation. (h–j) Electrochemical impedance spectroscopy analyses—including Nyquist, Bode-magnitude and Bode-phase plots—confirm reduced impedance and enhanced conductivity. (k,l) High-sensitivity piezoresponse amplitude and phase mappings further verify nanoscale electrical responsiveness of the microbeads. Mean values are shown and error bars represent ± s.d. (*n* = 3 per group), as analyzed using one-way ANOVA with Bonferroni correction.

The outer DOTAP (dioleoyl-3-trimethylammonium propane, D) layer was fabricated using an ethanol-based direct lipid mixing strategy, in which DOTAP was first dissolved in absolute ethanol without any auxiliary components and subsequently dried to form a thin lipid film. Upon rehydration in aqueous medium, the lipid spontaneously reorganized into dispersed fragments and vesicle-like assemblies (Figure S6). The rehydrated lipid film was briefly sonicated to promote the formation of smaller, unilamellar vesicle–like structures,^46,47^ as it has been reported that vesicle curvature and lamellarity modulate the exposure of charged headgroups, thereby influencing electrostatic interactions with anionic interfaces. Smaller unilamellar vesicles are therefore expected to provide stronger charge-mediated adhesion than multilamellar vesicles.^48^ When incubated with the PCB, the cationic DOTAP assemblies readily adhered to the anionic hydrogel surface through interactions with −SO_3_^–^ (PSS) and −COO^–^/–NH_2_ (GelMA) residues, forming a uniform positively charged coating (Figure 3d). The preferential localization of DOTAP at the periphery is consistent with electrostatic association, in which PCB carry negative charge whereas DOTAP is positively charged (Figure S7a). The average diameter of D@PCB was ≈130 μm (Figure 3e), a size well suited for injection and local tissue regeneration. Enzymatic degradation assays in collagenase-containing conditions demonstrated that both PCB and D@PCB were biodegradable within 2 weeks, simulating in vivo enzymatic activity and confirming their potential as resorbable scaffolds (Figure S7b).

### 2.3 Rheology of PCB

Regarding scaffold integration and the minimization of biological barriers at the brain-biomaterial interface, the storage modulus (*G*′) is a critical determinant of mechanical compatibility.^49^ The self-healing characteristics of MB, PCB, and D@PCB arise from reversible noncovalent interactions, which preserve material integrity under deformation and facilitate cellular infiltration and tissue remodeling.^50,51^ Following exposure to 100% strain, both *G*′ and the loss modulus (*G*″) rapidly recovered to their initial values across all formulations, underscoring their resilience under dynamic mechanical loading. Incorporation of conductive and bioactive nanocomponents in PCB and the additional DOTAP coating in D@PCB resulted in lower *G*′ values relative to MB, rendering these formulations more compliant and mechanically softer. This reduction is attributable to hydrated conductive domains and interfacial interactions introduced by the additional components, which modulate network stiffness while maintaining structural integrity. Importantly, MB, PCB, and D@PCB all exhibited storage moduli within the range reported for brain parenchyma,^52^ supporting their suitability as injectable scaffolds for neural repair (Figure 3f).

### 2.4 Electrochemical Charge Transport and Redox Behavior of PCB

To evaluate whether D@PCB can support alternating current flow under high-frequency stimulation, D@PCB were deposited onto conductive FTO substrates and subjected to an oscillating field. The resulting current–time responses confirmed that the microbeads form continuous conductive paths across the electrode. The observed current transients likely arise from complementary conduction mechanisms, with AuCDs facilitating interfacial electron transfer and PEDOT:PSS providing a continuous bulk network. Their integration supports the ability of the hybrid hydrogel to sustain conductive pathways relevant for externally driven stimulation (Figure 3g).^52,53^ Cyclic voltammetry further corroborated these findings (Figure S8a), whereas PCB and D@PCB exhibited pronounced capacitive loops. The larger enclosed area of the conductive formulations indicates enhanced charge storage capacity, consistent with the hybrid conduction pathways established by AuCDs and PEDOT:PSS.^54^

Electrochemical impedance spectroscopy (EIS) further resolved the charge-transport characteristics of the different microbead formulations. MB exhibited a near-vertical low-frequency branch without a discernible semicircle, consistent with a largely blocking capacitive interface.^55^ In contrast, PCB displayed a compressed semicircle with small real-axis intercepts, indicating low interfacial resistance and mixed capacitive behavior,^56^ while D@PCB preserved this low-impedance profile with only minor dispersion, suggesting that the cationic coating does not substantially hinder charge transfer at the hydrogel–electrolyte interface under small-signal interrogation (Figure 3h). Bode magnitude plots further showed frequency-independent impedance for PCB and D@PCB, characteristic of predominantly behavior (slope ≈ 0 on log–log axes), versus a steep rise for MB (|*Z*| ∝ *f* ^–1^), corroborating a capacitive interface (Figure 3i).^55,56^ Correspondingly, Bode phase showed angles approached 0° for PCB/D@PCB versus −80° to −90° for MB. These differences are expected when an electronic percolation network (e.g., PEDOT:PSS/Au-based pathways) is embedded within an ionically conducting hydrogel matrix (Figure 3j).^57^ Notably, both PCB and D@PCB maintained substantially lower impedance at 1 kHz—the frequency range relevant for neural recording and stimulation, maintaining mechanical compliance advantageous for brain tissue integration.^52^

Conductive AFM was used to probe the local electrical behavior of PCB and D@PCB (Figures 3k,l and S8b). Step-bias recordings on D@PCB showed a clear increase in current with applied voltage, and *I*–*V* sweeps exhibited quasi-linear trends within the tested range, consistent with effective charge transport through the conductive phase. The PCB displayed slightly broader separation between approach and retract traces in both amplitude and phase signals, whereas D@PCB showed smoother and more overlapping curves (Figure S8b). These observations suggest that surface modification with DOTAP slightly alters interfacial charge responses under bias.

### 2.5 HFMF-Driven Intracellular Transport Enhances Gene Delivery Efficiency of D@PCB

To minimize cytotoxicity while maintaining gene transfection efficiency, the formulations of the microbeads were optimized. After 24 h of incubation with NIH-3T3 fibroblasts, the viability of cells treated with MB, PCB, D@PCB, and D@PCB under HFMF stimulation remained above 85% (Figure 4a). Similar assays on the individual precursor components confirmed negligible cytotoxicity when AuCDs were used below 400 μg mL^–1^, PEDOT:PSS below 0.5%, and DOTAP below 800 μg mL^–1^; these concentrations were therefore applied in subsequent experiments (Figure S9). Confocal imaging further revealed not only sustained cell survival but also robust attachment and spreading on D@PCB, with or without HFMF, after 7 days of coculture (Figure 4b). Importantly, unlike specific metallic implants conventionally designed for magnetic hyperthermia,^58^ the AES is optimized for mixed ionic-electronic conduction. Under HFMF, the electromagnetic energy is primarily transduced into localized electrical cues rather than macroscopic thermal dissipation. As confirmed by real-time thermal monitoring (Figure S10), the temperature of the AES remained below 34 °C even after 5 min of continuous HFMF irradiation (exceeding our standard 3 min therapeutic duration). Because this peak temperature is strictly below the physiological threshold for heat-shock-induced cellular necrosis,^59^ this exceptional thermal safety profile is consistent with the high cellular viability observed in our in vitro models. This favorable compatibility is consistent with the intrinsic properties of GelMA hydrogels, which are widely recognized for their biocompatibility owing to preserved bioactive motifs such as RGD peptides and MMP-responsive sequences.^60^ Together, these results demonstrate that D@PCB maintain excellent cytocompatibility across conductive layer concentrations and under electric stimulation (Figures 4c and S11). Notably, D@PCB under HFMF stimulation exhibited enhanced cellular uptake of DOTAP, with fluorescence signals distributed throughout the cytoplasm.

**Figure 4:**
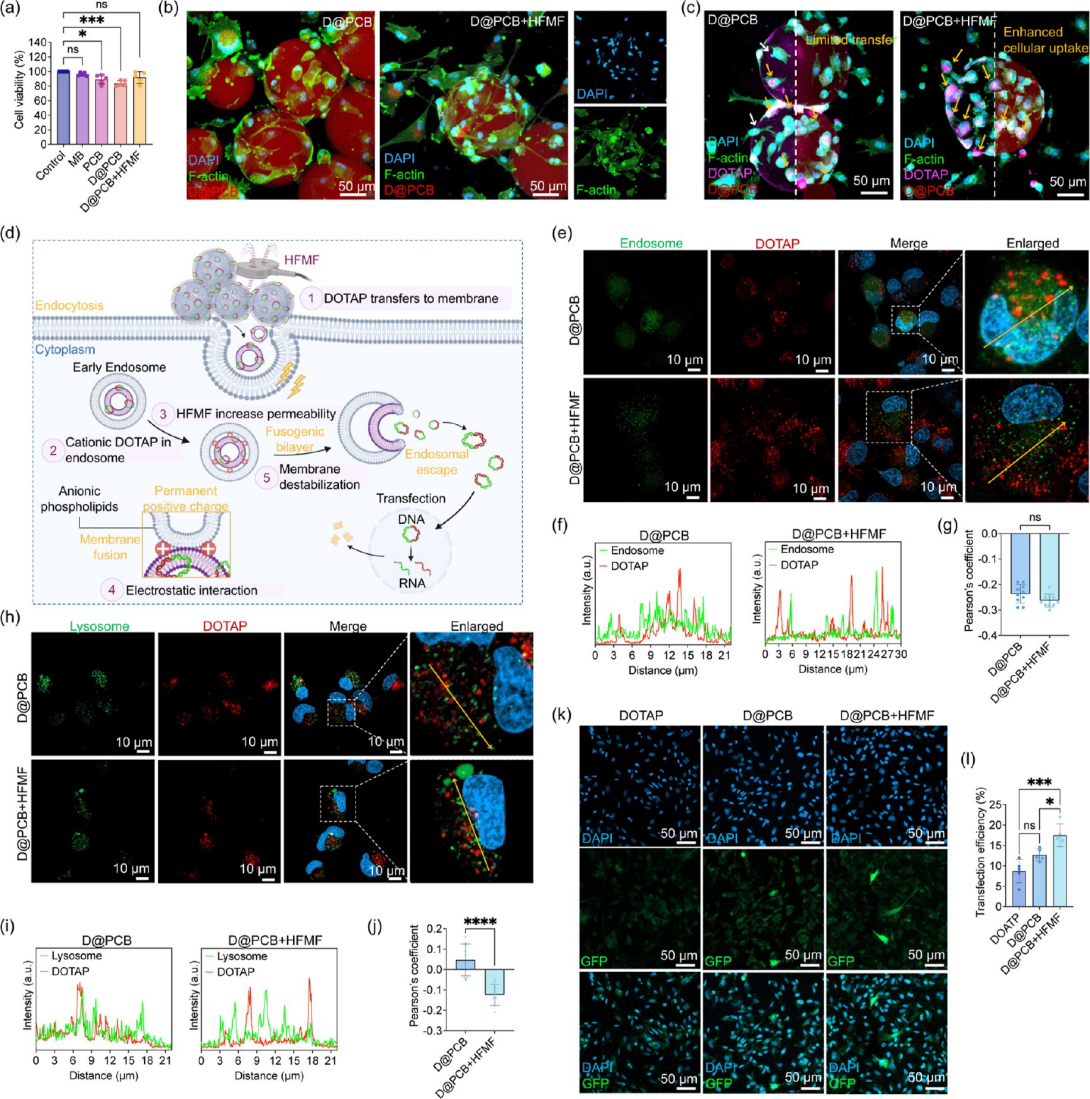
Cellular uptake, endosomal/lysosomal escape and transfection efficiency of D@PCB. (a) Cell viability of NIH-3T3 cells after incubation with different microbead formulations, showing negligible cytotoxicity (*n* = 5, mean ± s.d.; one-way ANOVA with Tukey’s test). (b) Confocal images of NIH-3T3 cocultures indicating that high-frequency magnetic-field stimulation promotes proliferation without toxicity in 7 days. (c) Cellular uptake at 24 h, demonstrating enhanced internalization under HFMF. (d) Schematic of DOTAP release from D@PCB and subsequent endosomal escape of gene cargo. (e–g) Endosome assay at 1 h: (e) confocal images showing colocalization of early endosomes (green) with DOTAP (red); (f) representative fluorescence line profiles; (g) Pearson’s correlation showing decreased endosomal retention with HFMF (*n* = 10, mean ± s.d.; one-way ANOVA with Tukey’s test). (h–j) Lysosome assay at 2 h: (h) confocal colocalization of LysoTracker (green) and DOTAP (red); (i) fluorescence line profiles; (j) Pearson’s coefficients indicating reduced lysosomal sequestration and enhanced escape with HFMF (*n* = 10, mean ± s.d.; one-way ANOVA with Tukey’s test). (k) CLSM images of NIH-3T3 cells transfected with GFP-DNA using DOTAP or D@PCB. (l) Transfection efficiency quantified across DOTAP and D@PCB groups ± HFMF (*n* = 5, mean ± s.d.; one-way ANOVA with Tukey’s test).

To evaluate the contribution of D@PCB to gene delivery, the mechanisms of cellular uptake and endosomal escape were investigated (Figure 4d). The delivery process is synergistically driven by both physical and chemical mechanisms. Under a high-frequency magnetic field (HFMF), responsive microbubbles (MBs) generate local electric fields that increase cell membrane permeability, while DOTAP detaches from the MBs to facilitate endocytosis.^61^ DOTAP, a benchmark cationic lipid for nonviral vectors, forms electrostatic complexes with nucleic acids. Owing to its permanent quaternary ammonium charge, DOTAP maintains its cationic nature within the acidic endosome. These cationic headgroups strongly interact with anionic phospholipids in the endosomal membrane to promote lipid mixing and fusogenic bilayer formation. This process destabilizes the membranes and releases the nucleic acids into the cytosol.^62^ Through this synergistic mechanism, the miR-6236 sponge delivered by D@PCB can efficiently reach the cytoplasm for functional activity.

Previous studies have reported that externally applied high-frequency fields acting on conductive or magnetically responsive matrices can induce local electrical currents that enhance intracellular uptake.^63^ In our system, such HFMF-driven electrical cues, together with the cationic nature of DOTAP, are expected to promote strong interactions between DOTAP-coated microbeads and endosomal membranes, thereby facilitating vesicular escape. To directly evaluate intracellular trafficking, confocal laser scanning microscopy (CLSM) imaging was performed with costaining of endosomal and lysosomal markers. After 1 h of incubation, CLSM images showed that D@PCB already exhibited partial escape from early endosomes, while HFMF stimulation further reduced colocalization (Figure 4e). Line-profile analysis confirmed less fluorescence overlap between D@PCB and endosomal signals under HFMF stimulation (Figures 4f and S12a). Quantitative analysis further supported this, revealing significantly decreased Pearson correlation values, which reflects a weaker overall intensity correlation between the nanoparticles and early endosomes, indicating reduced sequestration (Figure 4g). At 2 h, lysosomal tracking revealed a parallel trend. CLSM images and line-profile analyses showed more extensive lysosomal accumulation of D@PCB without stimulation, whereas HFMF stimulation reduced overlap with lysosomal markers (Figures 4h,i and S12b). Correspondingly, Pearson correlation values between DOTAP and lysosomes were markedly decreased under HFMF stimulation (Figure 4j). This demonstrates that electrical stimulation not only accelerates early endosomal escape but also effectively prevents subsequent lysosomal entrapment and degradation of the therapeutic cargo. Together, these results indicate that HFMF-assisted D@PCB provide a dual benefit for intracellular delivery: more rapid bypass of endosomal barriers and reduced lysosomal degradation, thereby maintaining the cytosolic availability of DOTAP–DNA complexes for transcriptional activity.

To validate the general DNA delivery capability of D@PCB, we performed a proof-of-concept transfection assay using a green fluorescent protein plasmid DNA (GFP pDNA) in NIH-3T3 fibroblasts and Neural stem cells (NSC). CLSM imaging confirmed robust intracellular GFP expression following incubation with D@PCB under HFMF stimulation (Figures 4k and S13). Quantification of fluorescence intensity further demonstrated significantly enhanced transfection efficiency in the HFMF-treated group compared to D@PCB without stimulation or DOTAP alone (Figure 4l). These results establish that the D@PCB system can effectively deliver and release functional plasmid DNA into cells and that high-frequency stimulation further augments this nonviral transfection.

### 2.6 Electroactive Microbeads Promote Neuronal Differentiation of NSCs under Electric Stimulation

Neural stem cells (NSCs) were harvested from E16 Wistar rat embryos and cocultured with the microbead formulations for differentiation assessment (Figure 5a). To evaluate the effect of electric stimulation, selected groups were subjected to a high-frequency magnetic field (HFMF; Power Cube 32/900, Honor Industries) at 2.24 kW and 1 MHz for 3 min. By day 7, CLSM images revealed evident neuronal differentiation and neurite extension across all groups (Figure 5b). The expression of the neuronal marker Tuj1 and the astroglial marker glial fibrillary acidic protein (GFAP) was analyzed to assess lineage-specific differentiation. Among all groups, D@PCB + HFMF treatment induced the most pronounced neurite sprouting, with a marked increase in Tuj1-positive neurons and no significant change in GFAP-positive cells (Figure 5c). Quantitative analysis confirmed that D@PCB alone already promoted neuronal differentiation relative to MB or PCB, while HFMF stimulation further amplified this effect without enhancing glial activation (Figure 5d). These results suggest that the conductive D@PCB, particularly under HFMF, provide an electroactive microenvironment that facilitates neuronal polarization and outgrowth.

**Figure 5:**
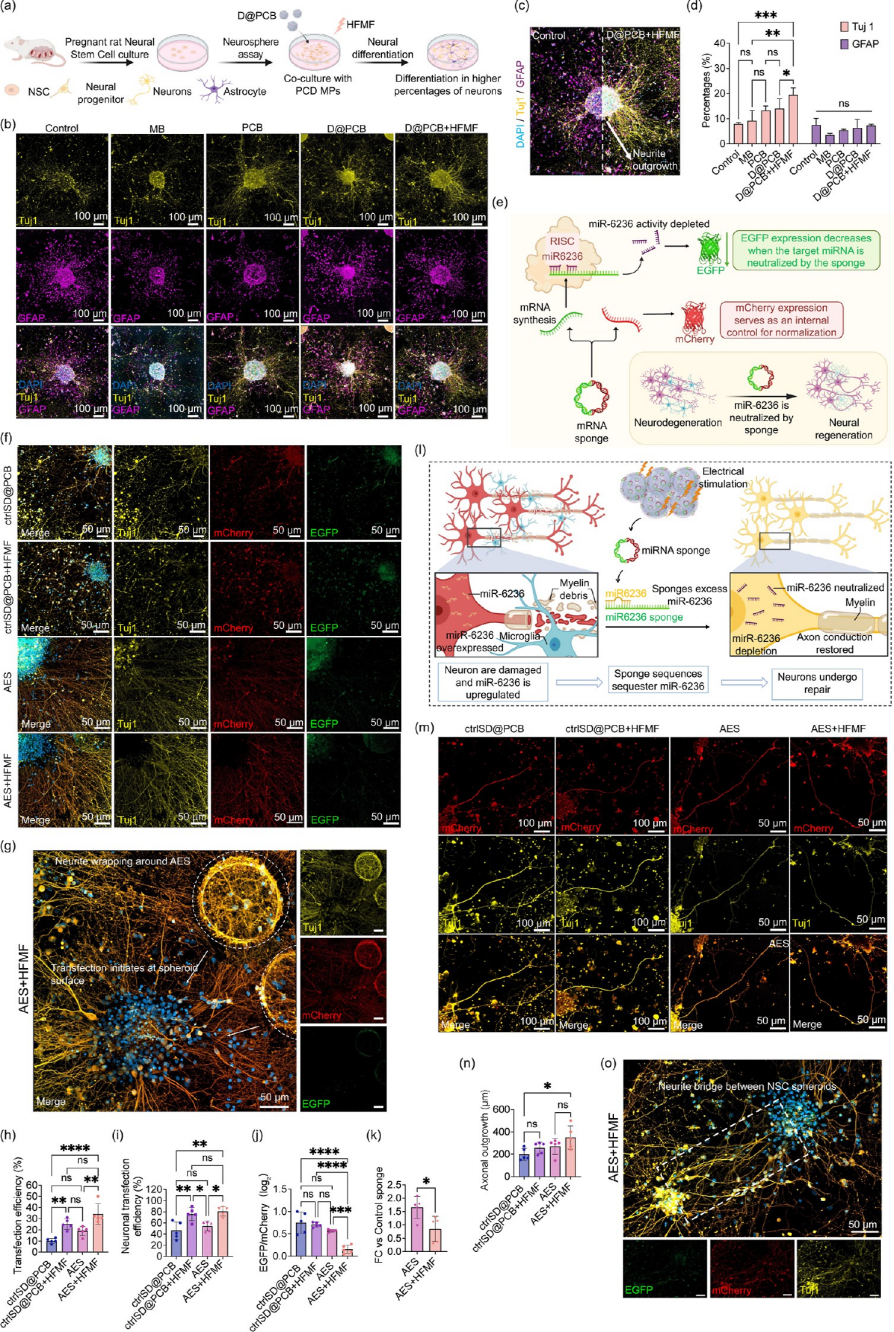
Neural differentiation, gene transfection and axonal regeneration mediated by miR-6236 sponge–loaded D@PCB. (a) Schematic workflow outlining neural stem cell isolation from embryonic mouse brains. (b) Representative CLMS images of NSCs after 7 days of differentiation showing βIII-tubulin-positive neurons (Tuj1, yellow) and glial fibrillary acidic protein (GFAP, purple)–positive astrocytes. (c) CLSM image of D@PCB-treated with HFMF cultures. (d) Quantitative analysis of neuronal versus astrocytic populations (*n* = 3, mean ± s.d.; *t*-test with multiple comparisons). (e) Mechanistic illustration of DOTAP-mediated plasmid delivery from D@PCB. (f) CLSM images comparing the fluorescence signals of control sponge (ctrlS) and miR-6236 sponge (miS) constructs demonstrated clear coexpression of mCherry and EGFP in neurons. (g) Spatial mapping of reporter expression revealed that transfection initiated at the spheroid surface. (h) Transfection efficiency quantified as the ratio of mCherry^+^ cells among all DAPI^+^ nuclei, and (i) neuronal transfection efficiency was defined as the fraction of mCherry^+^ cells within the Tuj1^+^ neuronal population. (j) The sensor ratio, expressed as log_2_(d2EGFP/mCherry) intensity within Tuj1^+^∩ mCherry^+^ neurons. (k) Fold-change analysis of the sensor ratio relative to the matched ctrlS baseline across four conditions (ctrlSD@PCB, ctrlSD@PCB + HFMF, AES and AES + HFMF; *n* = 5 fields per condition). (l) Schematic diagram summarizing the neuroregenerative mechanism. (m) Representative CLSM images of axonal projections in ctrlS and miS groups. (n) Quantification of axonal length in the AES + HFMF group (*n* = 5, mean ± s.d.; one-way ANOVA with Tukey’s test). (o) CLSM images illustrate continuous neurite bridges connecting adjacent NSC spheroids.

The enhanced neuronal differentiation observed with D@PCB, particularly under HFMF, can be attributed to the formation of an electroactive microenvironment around the microbeads. The PCB network supports mixed ionic–electronic transport, enabling subtle bioelectrical interactions at the cell–material interface that favors neuronal polarization and neurite outgrowth. When exposed to HFMF, the conductive microbeads are expected to experience inductively generated electrical cues, thereby providing wireless local stimulation to the surrounding NSCs. Meanwhile, the GelMA matrix preserves mechanical compliance and cell-adhesive motifs compatible with soft neural tissue. Together, these features help establish an instructive electrical microenvironment that promotes NSC differentiation toward a neuronal lineage while avoiding excessive glial activation.^64^

### 2.7 D@PCB for Ex Vivo Gene Transfection in Neural Stem Cells

Having demonstrated the endosomal escape capability of DOTAP under HFMF stimulation and the significantly enhanced transfection efficiency of D@PCB in NIH-3T3 cells, we next evaluated gene delivery performance in NSCs and their neuronal progeny. To achieve functional gene modulation, miR-6236-targeted sponges (miS)/D were loaded onto PCB (miSD@PCB, AES) and cocultured with NSCs. The miS plasmid is based on a previously reported miR-6236 sponge–sensor design in which miR-6236, a negative regulator of neuronal remodeling in inhibitory or injury-mimicking environments, is functionally sequestered by engineered binding sites. Each plasmid carries two strong promoters and dual fluorescent reporters: a destabilized enhanced green fluorescent protein (d2EGFP) with a short half-life for monitoring miRNA activity, and mCherry as an internal control for normalization. In this dual-reporter construct, d2EGFP is fused to a miR-6236-responsive 3′UTR containing tandem sponge sequences. Thus, when sponge transcripts are efficiently expressed and bind endogenous miR-6236, d2EGFP fluorescence is selectively suppressed while mCherry expression remains largely unchanged (Figure 5e). The same sponge 3′UTR simultaneously functions as a competitive decoy, depleting miR-6236 from its endogenous targets and thereby relieving repression of pro-regenerative gene networks. Therefore, a lower d2EGFP/mCherry ratio is interpreted as stronger miR-6236–sponge engagement and more effective functional neutralization of miR-6236, rather than a reduction in plasmid uptake or reporter expression.

CLSM imaging revealed the transfection of control sponge/D@PCB (crtlSD@PCB) and AES in primary neurons after 48 h of incubation (Figure 5f). The ctrlS plasmid encodes a CXCR4-based nontargeting sponge sequence previously validated to have no effect on neuronal morphology or polarization,^65^ and therefore serves as a negative control to confirm that the observed effects arise from miR-6236 depletion. Compared to the control group, neurons treated with AES exhibited diminished EGFP fluorescence, confirming successful miR-6236 sponge expression and target binding. In parallel, enhanced Tuj1 immunostaining was observed in neurons exposed to AES relative to the control sponge, suggesting that miR-6236 depletion promotes neuronal differentiation and cytoskeletal maturation. Notably, mCherry fluorescence was strongest at the spheroid interface and gradually extended along the surrounding neurites, indicating that transfection initiated at the microbead surface where DOTAP redistribution and membrane contact were most pronounced (Figures 5g and S14). This spatial pattern supports that electrically induced currents enhance local cellular uptake and facilitate neurite-associated gene transfer. The microbeads were surrounded by long, aligned neurites, indicating their strong neural compatibility and ability to present adhesive cues on the hydrogel surface. Under HFMF stimulation, neurons exhibited denser and longer neurites wrapping around the AES, suggesting that electrically induced electrical currents further enhance neuronal proliferation and interconnection. Such wireless stimulation can facilitate intracellular delivery and expedite electrical signal propagation across neuronal networks, ultimately strengthening neurite–neurite communication and reducing the interface stress and inflammatory responses often associated with conventional implanted electrodes.^66^

### 2.8 Quantitative Analyses of Delivery Efficiency from Sponge Activity

Having established qualitative evidence of neuronal transfection, we next performed quantitative analyses to disentangle delivery efficiency from sponge activity. Overall transfection, defined as the proportion of mCherry^+^ cells among all DAPI^+^ nuclei, confirmed that both ctrlSD@PCB and AES achieved effective nonviral delivery, with a moderate increase in overall mCherry^+^ fraction under HFMF (Figure 5h). Neuronal transfection, determined as the percentage of mCherry^+^ cells within the Tuj1^+^ population, mirrored the overall trend (Figure 5i), indicating that HFMF stimulation did not compromise lineage specificity and that most transfected cells were neurons. To assess miRNA–sponge activity, we quantified the log_2_ of median d2EGFP/mCherry within Tuj1^+^∩mCherry^+^ neurons in each field. Because d2EGFP is fused to the sponge, its fluorescence decreases when endogenous miR-6236 binds, such that lower log_2_ values reflect stronger miRNA engagement (Figure 5j). Compared with the control sponge, the miR-6236 sponge yielded consistently lower EGFP/mCherry ratios, confirming effective target sequestration. Under HFMF, a further downward shift was observed in several fields, suggesting that electric stimulation enhances endosomal escape or cytoplasmic accessibility of the sponge, thereby increasing functional interaction with miR-6236. To compare activity across physical contexts, we expressed the results as a fold-change (FC) of median d2EGFP/mCherry in the miR-6236 sponge relative to the matched control sponge baseline (geometric mean of ctrlSD@PCB for without HFMF and ctrlSD@PCB for with HFMF) (Figure 5k). In this representation, FC ≈ 1 denotes parity with the control, FC > 1 indicates reduced miR-6236 engagement, and FC < 1 indicates stronger miR-6236 capture by the sponge. In the absence of HFMF, the miR-6236 sponge exhibited slightly elevated FC values, suggesting that while the plasmid was efficiently expressed, a portion of the sponge transcripts may not yet have achieved full cytoplasmic accessibility or interaction with endogenous miR-6236. Under HFMF stimulation, FC values shifted downward toward or below unity, consistent with enhanced sponge–miRNA binding once electric stimulation promoted endosomal release and cytoplasmic redistribution.

To further elucidate the functional consequence of miR-6236 depletion, we examined neuronal morphology and network organization in transfected NSC cultures. The schematic illustration depicts that in injured neuronal microenvironments, elevated miR-6236 suppresses cytoskeletal gene expression and disrupts axonal conduction, whereas sponge-mediated sequestration of miR-6236 restores neurite outgrowth and axonal integrity (Figure 5l). Consistent with these phenotypic observations, Figure S15 presents a mechanistic working model in which sponge-mediated sequestration of miR-6236 is proposed to relieve repression of pro-regenerative transcriptomic programs, thereby restoring microtubule organization and stabilizing the cytoskeletal framework. This interpretation is supported by prior evidence that miR-6236 negatively regulates neuronal morphogenesis and regeneration,^24^ by its independent identification in a TBI-associated brain-derived extracellular vesicle miRNA biomarker panel,^25^ and by the broader concept that CNS injury-associated miRNAs function through interconnected repair-related pathways rather than isolated single-gene events.^67^ Consistent with this model, confocal imaging revealed that neurons treated with AES exhibited markedly elongated and highly oriented neurites compared with those receiving the control sponge (Figure 5m). When stimulated under HFMF, neurite extension was further enhanced, producing extensive, aligned Tuj1^+^ fibers radiating from spheroid surfaces. Quantitative analysis confirmed a significant increase in average neurite length and network density in the AES group relative to ctrlSD@PCB (Figure 5n), indicating that electrically electrical cues synergize with gene modulation to potentiate axonal elongation. Moreover, long, continuous neurite bundles were observed bridging neighboring NSC spheroids (Figure 5o), suggesting that miR-6236 depletion and electric stimulation together promote interspheroid connectivity and the formation of axon-like conduits between neuronal clusters. These results demonstrate that sponge-mediated miR-6236 neutralization effectively reactivates intrinsic neuronal growth programs, while electrical stimulation further reinforces structural integration across the developing neural network.

### 2.9 In Vivo Transfection of AES in a Mouse TBI Model

Seven-week-old female C57BL/6 mice (*n* = 5 per group) were used for in vivo evaluation of AES. The overall experimental schedule, including microbead implantation and subsequent electric stimulation (Figure 6a). A TBI was induced in the motor cortex using a 2 mm flat-end biopsy punch to a depth of approximately 1.5 mm. Immediately after tissue removal, the lesion cavity was filled with AES (Figure 6b). HFMF stimulation began 24 h postsurgery and was applied once daily. For short-term transfection analysis, animals were sacrificed on day 7; for long-term neuroregenerative assessment, HFMF exposure continued until day 14, and brains were harvested at later time points for histological and immunofluorescence analyses.

**Figure 6:**
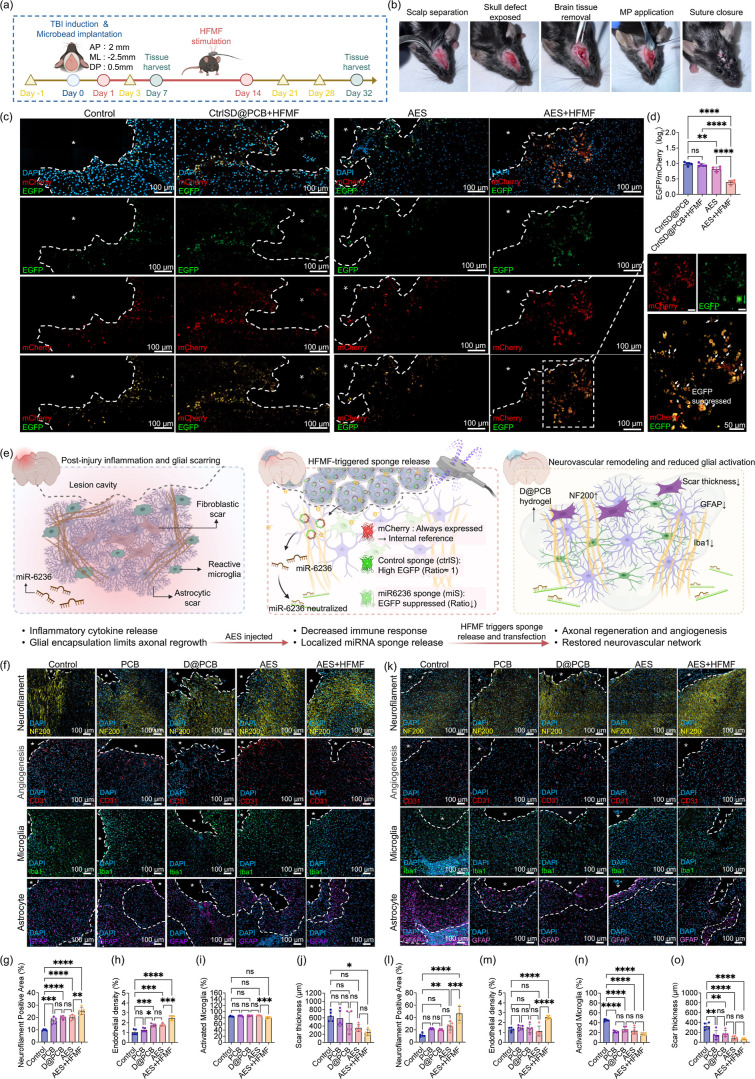
In vivo transfection and neuroregenerative effects of AES in a TBI model. (a) Experimental timeline showing TBI induction, microbead implantation and daily HFMF stimulation (7- or 14-day regimens), followed by sacrifice for analysis. (b) Representative surgical images of lesion creation, particle injection and wound closure in motor cortex. (c) In vivo transfection procedure and CLSM images around the TBI cavity at day 7; reduced EGFP (green) relative to mCherry (red) indicates efficient miR-6236 depletion and target repression. Asterisks mark the cavity margin. (d) Quantification of transfection efficiencies with and without HFMF (*n* = 5, mean ± s.d.; one-way ANOVA with Tukey’s test). (e) After TBI, excessive inflammatory cytokine release and glial encapsulation form dense astrocytic and fibroblastic scars, disrupting tissue integrity and inhibiting axonal regrowth. Upon microsphere injection and HFMF stimulation, local electrical cues from D@PCB induce DOTAP detachment and controlled release of the miR-6236 sponge, which neutralizes endogenous miR-6236 and suppresses EGFP reporter expression. (f) Peri-lesional immunostaining at day 7 showing neurons (NF200, yellow), endothelial cells (CD31, red), microglia/macrophages (Iba1, green) and astrocytes (GFAP, purple), with DAPI (blue) marking nuclei. (g–j) Quantitative analyses of NF200^+^ area, endothelial density, activated microglia and glial-scar thickness (*n* = 5, mean ± s.d.; one-way ANOVA with Tukey’s test). (k) Peri-lesional immunostaining at day 32 demonstrating persistent neuronal and vascular restoration and attenuated gliosis. (l–o) Quantification at day 32 of NF200^+^ area, endothelial density, activated microglia and scar thickness (*n* = 5, mean ± s.d.; one-way ANOVA with Tukey’s test).

A pilot in vivo transfection experiment using an GFP-only plasmid demonstrated the intrinsic gene delivery capability of the AES (Figure S16), animals receiving HFMF stimulation exhibited markedly stronger fluorescence at the implantation site compared with nonstimulated controls, confirming electrically enhanced DNA transfection efficiency. Building upon this result, the implantation of AES followed by HFMF stimulation enabled efficient in vivo transfection of the miS within the injured cortex. Confocal images obtained 7 days post-treatment revealed strong reporter expression around the lesion cavity (Figure 6c). Both ctrlS and miS-targeted sponge plasmids were successfully delivered using the AES platform. In magnified regions, EGFP fluorescence was markedly reduced relative to mCherry, indicating effective sequestration of endogenous miR-6236 and suppression of its target reporter. The constant red signal confirmed stable expression from the internal control promoter. Quantitative analysis further demonstrated that HFMF exposure during treatment enhanced gene-transfer efficiency, as reflected by a significantly lower EGFP/mCherry fluorescence ratio compared with nonstimulated samples-showing a 1.5-fold reduction relative to the nonstimulated group (Figures 6d and S17). HFMF significantly increased the fraction of mCherry^+^ cells, indicating enhanced gene transfer efficiency, while miR-6236 sponge constructs consistently displayed lower d2EGFP/mCherry ratios than control sponges, consistent with stronger target engagement. This electrical stimulation likely promoted transient membrane depolarization and facilitated endosomal escape, thereby improving intracellular delivery of genetic cargo.

### 2.10 Regeneration of Neurovascular Tissues and Modulation of the Glial Microenvironment

TBI initiates a cascade of pathological responses, including the rapid activation of resident immune and glial cells at the lesion interface.^68^ While this early inflammatory surge serves to limit secondary damage by clearing cellular debris and restricting the spread of injury, persistent or excessive activation often triggers the formation of a dense glial scar and fosters a neurotoxic microenvironment. This maladaptive response can hinder neuronal regeneration by disrupting the proliferation and differentiation of neural stem cells, ultimately compromising recovery.

To illustrate the overall mechanism of in vivo gene transfection and subsequent repair outcomes, a schematic diagram (Figure 6e) summarizes the spatiotemporal events following D@PCB microbead implantation and HFMF stimulation. Immediately after traumatic injury, the lesion core is dominated by inflammatory cytokine release and glial activation. Upon local administration of AES, the conductive microbeads generate local electric cues under HFMF stimulation, promoting the release and intracellular uptake of miR-6236 sponge plasmids.

The dual-reporter design of the sponge construct allows real-time visualization of transfection and miRNA activity in vivo: EGFP serves as a miR-6236-responsive reporter, while mCherry functions as a stable normalization control driven by an independent promoter. In tissues receiving the control sponge (ctrlS), both fluorescent proteins are robustly expressed, yielding bright green (EGFP) and red (mCherry) signals with an approximately constant EGFP/mCherry ratio (∼1). In contrast, delivery of the miR-6236 sponge (miS) results in binding and sequestration of endogenous miR-6236, thereby suppressing EGFP expression while maintaining mCherry fluorescence. The observed decrease in the EGFP/mCherry ratio thus reflects successful miRNA neutralization.

When combined with HFMF stimulation, the local electric field further enhances plasmid release, membrane permeability, and transcriptional activation, producing stronger transfection efficiency and more pronounced EGFP downregulation near the lesion site. This enhanced miR-6236–sponge interaction leads to reduced glial reactivity and a transition toward a pro-regenerative microenvironment. In the subsequent repair phase, brain tissues treated with AES + HFMF display thinner glial scars, lower GFAP and Iba1 levels, and increased NF200 and CD31 expression, indicative of attenuated astrogliosis and microgliosis alongside improved axonal and vascular regeneration.

### 2.11 In Vivo Regeneration of Neuron and Blood Vessels at Day 7, 32 and 67

To evaluate the short-term effects of AES on neural regeneration and immune modulation, brain sections were analyzed 7 days after implantation across five groups: untreated control, PCB, D@PCB, AES, and AES with HFMF stimulation (Figure 6f). CLSM of NF200 staining revealed sparse axonal structures near the lesion border in the control group, whereas microbead-treated groups exhibited a gradual restoration of axonal networks. Notably, animals receiving AES + HFMF displayed dense, elongated NF200-positive fibers (Figure 6f), suggesting improved axonal guidance and plasticity. Quantitatively, neurofilament-positive area was significantly elevated in this group (Figure 6g). In parallel, CD31 staining showed a substantial increase in microvascular density within the lesion vicinity, particularly in the AES + HFMF group, with more organized and branched endothelial networks visible under CLSM (Figure 6f). The quantitative analysis revealed over a 2-fold increase in CD31^+^ area relative to untreated tissue (Figure 6h), highlighting the angiogenic synergy between conductive stimulation and miR-6236 modulation. Importantly, the percentage of activated microglia (Iba1^+^ cells) remained statistically unchanged across all groups (Figure 6i). The minimal variation in Iba1 expression underscores the immune compatibility of the system and suggests that the therapeutic intervention avoids excessive pro-inflammatory activation. Furthermore, GFAP staining demonstrated a marked attenuation of astroglial scarring in the AES + HFMF group, with confocal images showing narrower GFAP-positive boundaries and reduced scar encapsulation along the cavity edge (Figure 6f). This was quantitatively supported by a significant decrease in glial scar thickness (Figure 6j), highlighting the formulation’s ability to mitigate chronic glial barrier formation. These data suggest that AES support a pro-regenerative microenvironment by enhancing axon and vessel recovery while limiting astrocytic encapsulation and microglial activation.

At 32 days postinjury, chronic tissue remodeling was evaluated to determine whether the therapeutic effects of the AES system persisted beyond the acute phase (Figure 6k). CLSM imaging revealed robust NF200+ axonal bundles extending from the lesion edge in the AES + HFMF group, with more linear and directionally aligned trajectories compared to other groups (Figure 6k). This organized axonal morphology was consistent with sustained neuroregeneration and was accompanied by significantly higher NF200^+^ area relative to all other conditions (Figure 6l). CD31 staining further showed extensive vascular networks around the lesion in the AES + HFMF group, suggesting ongoing angiogenesis and vessel maturation, with a ∼2.5-fold increase in CD31^+^ signal compared to control (Figure 6m). In contrast, quantification of Iba1 immunoreactivity showed no significant differences across groups, indicating that the implanted conductive hydrogel microbeads did not induce long-term neuroinflammation (Figure 6n). Interestingly, in the untreated control brains, a densely packed DAPI^+^ region was observed adjacent to the lesion in both Iba1 and GFAP staining channels. This region, while exhibiting intense nuclear staining, showed minimal overlap with either microglial or astrocytic markers, suggesting the presence of a nonglial, possibly undifferentiated or degenerative cell population. In contrast, GFAP staining in the AES + HFMF group showed a markedly thinner and more confined glial scar compared to all other groups, indicating effective suppression of reactive astrogliosis and glial encapsulation (Figure 6o). Collectively, these results demonstrate that AES with HFMF stimulation not only promote sustained axonal and vascular remodeling, but also prevent long-term glial scarring and abnormal cell aggregation near the injury core, facilitating a more permissive microenvironment for functional recovery.

To further evaluate the durability and sustained regenerative efficacy of the AES platform in the chronic phase of traumatic brain injury, we assessed the local neural microenvironment at 67 days postinjury (Figures S18 and S19). Immunofluorescence staining showed that the therapeutic effects of AES coupled with HFMF stimulation were robustly maintained over this extended period. The AES + HFMF group exhibited a dense, highly organized NF200-positive axonal network alongside sustained CD31-positive microvascular density, significantly outperforming the untreated control and other monotherapy groups.

Regarding the neuroinflammatory response, Iba1 staining revealed a slight elevation in microglial activation in the base PCB scaffold group compared to the untreated control. This localized increase is consistent with a host response to the long-term physical presence of foreign synthetic materials within the brain parenchyma. Notably, the integration of the complete AES platform coupled with HFMF stimulation effectively mitigated this immune response. The AES + HFMF group maintained consistently low levels of activated Iba1-positive cells, suggesting minimal chronic neuroinflammation and no overt evidence of late-stage foreign body encapsulation. To further assess the chronic inflammatory phenotype, additional immunofluorescence staining for iNOS and CD206 was performed (Figure S19a–c). Compared with untreated and material-only groups, AES + HFMF treatment showed reduced iNOS-positive inflammatory signals together with elevated CD206-positive reparative signals, indicating a favorable shift in chronic macrophage/microglia polarization. This result is consistent with the reduced Iba1/GFAP reactivity observed in the same animals and supports the conclusion that AES fosters a more regeneration-permissive immune microenvironment over the long-term. This observation is also consistent with previous literature demonstrating that mechanically compliant, electrically active bioelectronic interfaces can attenuate chronic neuroinflammatory responses.^69^ Since damage-associated molecular patterns (DAMPs) released from degenerating neurons are recognized drivers of sustained neuroinflammation after TBI, this chronic immune improvement is likely a secondary consequence of successful neuronal rescue rather than direct immune-cell reprogramming.^70^ By effectively neutralizing miR-6236 to stabilize the neuronal cytoskeleton and prevent progressive axonal degeneration, our intervention may reduce the release of these pro-inflammatory DAMPs, thereby limiting the secondary inflammatory cascade and fostering a chronic immune microenvironment more conducive to long-term regeneration.^70,71^

Alongside microglial attenuation, GFAP staining revealed that AES + HFMF treatment effectively prevented the consolidation of a dense long-term glial scar, displaying a significantly thinner astroglial boundary compared to control. Since sever tissue or thermal injury typically triggers robust reactive astrogliosis and dense glial scarring,^72^ the profound attenuation of these markers in the attenuation of these markers in the AES + HFMF group argues against overt local hyperthermia. The absence of thermal injury-associated pathology supports that the pro-regenerative efficacy is driven by targeted electrical stimulation, ensuring exceptional tissue safety.

Collectively, these 67-day results suggest that the synergistic effect of the AES and HFMF stimulation not only accelerates acute/subacute tissue repair but also helps maintain a microenvironment permissive for long-term regeneration.

### 2.12 MRI-Based Diffusion Tensor Imaging Fiber Tracking of Lesion-Related Motor and Sensorimotor Pathways

To delineate the structural context of TBI and its impact on major sensorimotor circuits, MRI-based diffusion tensor imaging (DTI) tractography was conducted with anatomical segmentation of the primary motor cortex (M1), caudate putamen (CPU), thalamus (TH), and hindlimb somatosensory cortex (S1HL) (Figure 7a). These regions constitute key components of the cortico-thalamic and cortico-striatal loops that govern voluntary movement, sensory feedback, and motor learning.^73–75^ The spatial relationship among these regions is further illustrated in Figure 7c, showing the lesion site localized in the left M1 adjacent to CPU and S1HL, which are commonly affected in motor coordination deficits after cortical injury.^76^

**Figure 7:**
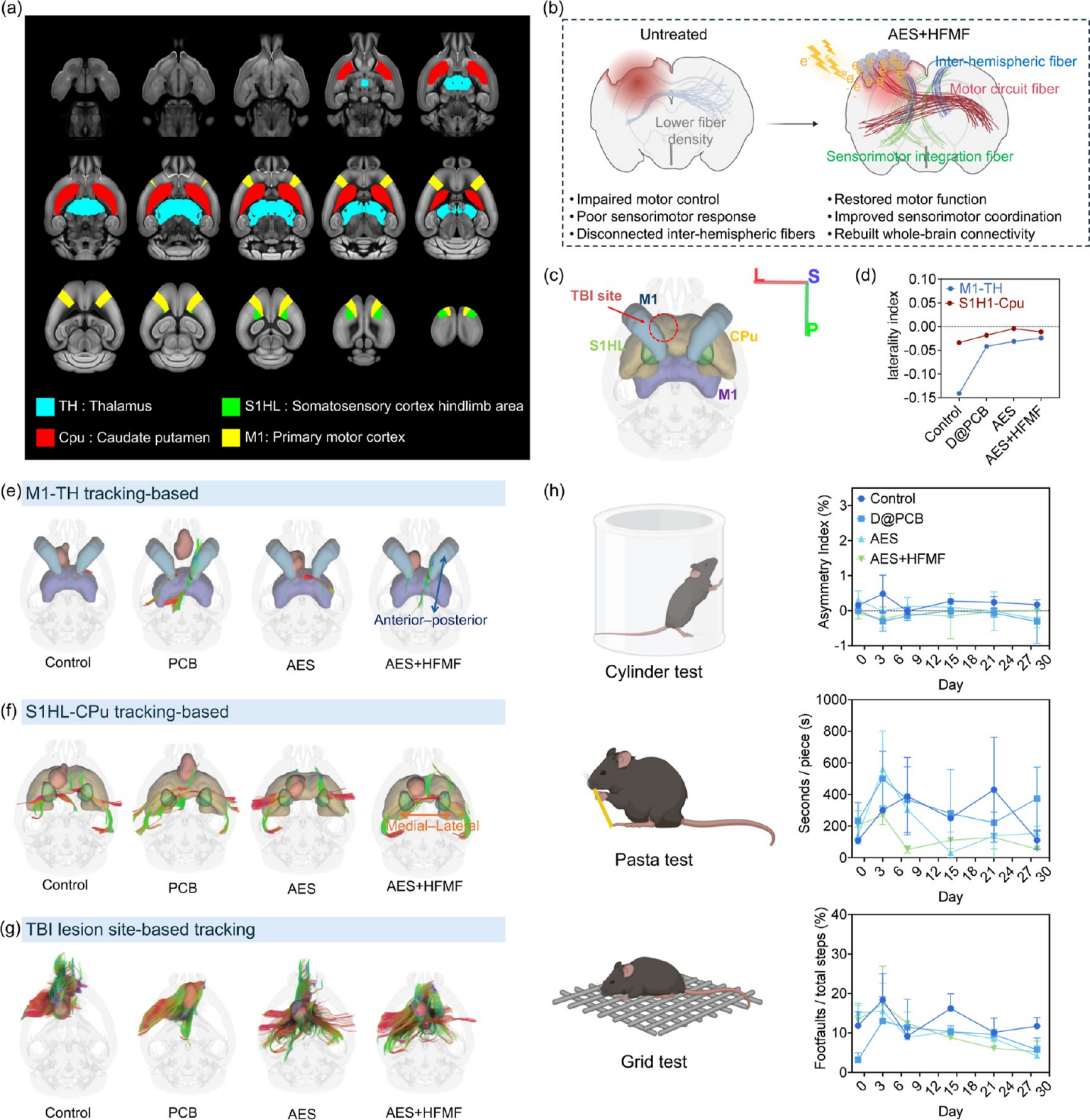
MRI fiber-tracking analysis and behavioral outcomes following D@PCB-based therapy after TBI. (a) Anatomical parcellation of major regions including thalamus (TH, cyan), caudate putamen (CPU, red), primary motor cortex (M1, yellow) and somatosensory hindlimb cortex (S1HL, green). (b) Schematic of the tractography approach illustrating reduced peri-lesional tract density. (c) Region-of-interest map indicating lesion location relative to M1, CPU and S1HL. (d) Quantification of fiber density in lesioned versus contralateral hemispheres showing partial recovery with AES + HFMF. (e) M1–TH pathway reconstructions revealing improved corticothalamic connectivity. (f) S1HL–CPU tracking of sensorimotor integration after treatment. (g) Lesion-centered whole-brain tractography confirming restoration of local and long-range projections. (h) Behavioral testing (grid-walk, cylinder and adhesive-removal) demonstrating improved motor coordination and sensorimotor function with therapy (*n* = 3, mean ± s.d.; one-way ANOVA with Tukey’s test).

DTI was performed at day 14 postinjury, corresponding to the subacute phase of TBI when acute edema and inflammatory artifacts have largely subsided, allowing reliable visualization of microstructural reorganization. This time point bridges our immunofluorescence observations at day 7 (acute inflammation) and day 32 (neuronal remodeling), thereby capturing the early stage of axonal sprouting and tract reformation while avoiding signal distortion from acute swelling. A schematic overview (Figure 7b) depicts the typical loss of corticothalamic and corticostriatal fibers following TBI, resulting in disrupted interhemispheric communication and weakened sensorimotor integration. After implantation of the conductive microbeads and application of HFMF stimulation, these networks were hypothesized to undergo partial restoration, enabling the re-establishment of both motor circuit and sensory feedback pathways. Based on this rationale, three tracking analyses were designed for evaluation: M1–TH (motor feedback pathway), S1HL–CPU (sensorimotor integration pathway), and TBI lesion site–based tracking (global network recovery).^76–78^ These parameters collectively provide an integrated view of how local cortical repair translates into long-range axonal reconnection and functional network recovery after injury.

To further examine the restoration of long-range connectivity, the three tracking analyses were reconstructed based on the major projection pathways. The M1–TH tracking-based reconstruction (Figures 7e and S20a) demonstrated that in the normal brain, corticothalamic fibers project along an anterior–posterior axis connecting the primary motor cortex to the thalamus.^79^ After TBI, this pathway showed marked disruption near the ipsilateral hemisphere, whereas the treated group exhibited partially restored fiber continuity and density across the lesion boundary, suggesting re-establishment of motor feedback transmission. The S1HL–CPU tracking-based reconstruction (Figures 7f and S20b) revealed that sensorimotor fibers connecting the hindlimb somatosensory cortex to the caudate putamen were oriented mainly along the medial–lateral axis.^80^ Postinjury disorganization of these fibers was evident, while treatment promoted better alignment and tract integrity, indicating enhanced sensorimotor integration and coordination.

Finally, TBI lesion site–based tracking (Figures 7g and S20c) was applied to visualize global fiber reorganization originating from the cortical injury site. In the untreated brain, tract density was sparse around the lesion core, whereas after treatment, more extensive fiber projections spanning both hemispheres and subcortical regions were observed, highlighting broad network restoration and axonal regrowth consistent with structural recovery seen in previous MRI-based regeneration studies.

In addition, a laterality index (LI) was computed for each pathway (Figure 7d) to assess hemispheric asymmetry in tract restoration. A higher LI toward the lesioned side indicates preferential reinnervation within the injured hemisphere, reflecting more robust local reconnection rather than contralateral compensation. In our data, the M1–TH pathway showed a significantly greater LI increase post-treatment compared to the S1HL–CPU pathway, further supporting prioritized recovery of motor feedback circuitry.

### 2.13 Animal Behavior Tests

To evaluate the functional recovery induced by the treatment, a series of behavioral assessments were conducted in female C57BL/6 mice (7 weeks old) subjected to TBI. The animals were divided into four groups (*n* = 3 per group): (1) control, (2) D@PCB, (3) AES, and (4) AES + HFMF. Behavioral testing was performed weekly for 32 days to monitor motor coordination and forelimb function over time (Figure 7h). No significant difference in body weight was observed among groups, indicating that all mice maintained stable physiological conditions throughout the experiment.

Motor asymmetry was first assessed using the cylinder test, which measures spontaneous forelimb use during rearing. Because the lesion was localized in the left motor cortex, mice initially exhibited reduced use of the contralateral (right) limb. Over time, the treated groups showed a gradual normalization of limb use, approaching bilateral balance, whereas the Sham and untreated groups maintained clear asymmetry. To further examine locomotor coordination, the grid-walking test was performed to quantify hind-limb foot-faults. The Sham group displayed the highest incidence of foot-slips, while the treated groups exhibited a lower and steadily declining error rate, suggesting improved motor coordination. Fine motor skills were subsequently evaluated using the pasta-handling test, which assesses forepaw dexterity and grasping ability. Untreated mice required longer times to complete the task, whereas the treated group completed it more rapidly by day 30, showing better fine-motor control. Although variability existed across individuals, the overall behavioral trends indicated that the treatment facilitated a more symmetrical and coordinated use of both forelimbs, consistent with the enhanced structural connectivity observed in the DTI analysis.

### 2.14 Long-Term Systemic Biosafety of AES Treatment

To evaluate the long-term in vivo biocompatibility and systemic biosafety of the AES system, histological examinations of major organs were performed. At 67 days postimplantation, major organs including the heart, liver, spleen, lungs, and kidneys were harvested from the treated mice and subjected to hematoxylin and eosin (H&E) staining (Figure S21). Compared to the control group, mice treated with AES and HFMF stimulation exhibited no observable morphological abnormalities, cellular necrosis, or inflammatory lesion formations in any of the examined organs. These results comprehensively confirm that the localized AES implantation and the remotely applied HFMF induce no severe systemic toxicity, highlighting the excellent long-term biosafety and clinical translational potential of this wireless bioelectronic platform.

## 3 Conclusion

In this study, we developed a wirelessly responsive, an adaptable electronic scaffold (AES) that couples nonviral miRNA modulation with localized electrical stimulation for neuroregeneration. The hybrid PEDOT:PSS–AuCDs conductive network enabled electrical responsiveness under HFMF stimulation, while the DOTAP coating supported efficient loading and intracellular delivery of miR-6236 sponges. This integrated design enhances functional attenuation of maladaptive miR-6236 activity and is associated with increased expression of regenerative markers, including enhanced neuronal differentiation, axonal extension, and reduced glial activation after traumatic brain injury.

Compared with conventional viral vectors and static conductive scaffolds, this strategy provides spatially controlled delivery and externally tunable stimulation. Previous electric field–assisted systems have improved intracellular uptake through physical perturbation but often lacked biocompatibility or sustained conductivity in hydrated environments.^81^ In contrast, AES maintains mixed ionic–electronic conduction in a fully hydrated state, supporting stable current propagation through the GelMA matrix. The ultrasmall carbon-based AuCDs mitigate the aggregation and cytotoxicity commonly associated with metallic nanoparticles while improving dispersion stability and charge-transfer capacity. Under HFMF, the electrical coupling of the composite is expected to generate local electrical cues that, together with DOTAP, facilitate increased uptake and endosomal escape of miR-6236 sponge constructs, consistent with our in vitro observations of enhanced internalization and lysosomal escape. Rather than relying on viral transduction, this multicomponent system substantially improves nonviral transduction efficiency relative to nonconductive or nonstimulated control, providing a wireless route to modulate miRNA activity in neural cells. Crucially, because the delivered plasmid DNA is a conventional nonviral construct lacking dedicated integration machinery, it is expected to remain predominantly as an extrachromosomal episome. Accordingly, the risk of insertional mutagenesis is considered substantially lower than that associated with integrating viral vectors.^82,83^ This transient expression profile is also well aligned with the therapeutic goal of temporary intervention during the acute and subacute phases of traumatic brain injury.

Beyond direct gene delivery, the electroactive microenvironment created by the microbeads further influenced neural differentiation outcomes. The conductive interface supported neuronal polarization and dense neurite interconnections, in line with report that electrical microcurrents and Ca^2+^-dependent signaling govern stem-cell-to-neuron transitions.^84^ Importantly, our in vitro and in vivo results show that the system consistently promotes Tuj1-positive neuronal differentiation without increasing GFAP expression, suggesting that the applied electrical cues remain within a physiologically compatible range that favors neurogenesis without exacerbating astroglia activation. These findings highlight the potential of combining molecular specificity with electrical functionality to promote coordinated neurogenesis and angiogenesis within injured cortical tissue. Moreover, extending this platform to multiplexed miRNA targets or to other neurodegenerative and neurotrauma contexts, such as Parkinson’s disease or spinal cord injury, could further broaden its therapeutic applicability.

While the GelMA-based bulk matrix of the AES demonstrates favorable biodegradability, caution must be exercised regarding the long-term fate of the embedded conductive components, specifically PEDOT:PSS and AuCDs. In principle, the ultrasmall size of the AuCDs (average diameter ∼2.6 nm) falls well below the critical ∼5.5 nm hydrodynamic diameter threshold established for efficient renal filtration and urinary excretion of inorganic nanoparticles. Such a renal–clearable profile is expected to significantly mitigate the risks of chronic systemic accumulation and associated nanotoxicity.^85,86^ Importantly, previous studies have demonstrated that PEDOT:PSS-based neural interfaces exhibit stable chronic biointegration and long-term electrochemical and biological stability, while related PEDOT-based conductive coatings have shown stable chronic neural recording performance for up to 540 days in the rat cortex.^87,88^ Consistent with these reports, our 67-day in vivo assessments showed no severe systemic toxicity or chronic foreign body response to the remaining PEDOT:PSS-containing conductive network. However, this 67-day observation window may not fully capture potential late-onset effects. Therefore, comprehensive pharmacokinetic tracking and chronic toxicity profiling over extended periods represent important next steps for the future clinical translation of this bioelectronic platform.

Overall, this study delineates a framework for wireless, molecularly targeted neuroregeneration. By integrating externally controllable electrical stimulation with miRNA network reprogramming in a soft, brain-mimetic hydrogel scaffold, AES provides a versatile foundation for next-generation bioelectronic therapies that bridge electrical control and genetic regulation to support functional recovery of the injured brain.

## 4 Experimental Section

### 4.1 Synthesis of Gold–Carbon Nanocomposites

Gold–carbon nanocomposites (AuCDs) were synthesized following a modified glutathione-mediated reduction protocol. Briefly, 1.2 mL of 100 mM l-glutathione (GSH, Sigma-Aldrich) was added to 4 mL of 20 mM HAuCl_4_ (chloroauric acid trihydrate, Sigma-Aldrich) under continuous stirring at 25 °C. The reaction mixture was stirred for 1 min until the yellow solution turned colorless, indicating the formation of Au–thiolate intermediates. Subsequently, 34.8 mL of deionized water was introduced, and the solution was maintained at 70 °C under gentle agitation (≈500 rpm) for 24 h. The reaction yielded a homogeneous aqueous dispersion exhibiting strong orange fluorescence, corresponding to the formation of AuCDs.

### 4.2 Synthesis of GelMA Hydrogels

Gelatin methacrylate (GelMA) was prepared according to a modified protocol. Type B bovine-skin gelatin (5 g, 10% w/v; Sigma-Aldrich) was dissolved in 50 mL of deionized water at 50 °C under constant stirring. Methacrylic anhydride (MA, Sigma-Aldrich) (2.5 mL) was then added dropwise, and the reaction proceeded for 3 h. The solution was dialyzed (12–14 kDa cutoff) against deionized water at 30 °C for 5 days, with water replaced twice daily to remove unreacted reagents and salts. The dialyzed product was lyophilized for 3 days to obtain a porous, foam-like GelMA, which was stored at −20 °C until further use.

### 4.3 Fabrication of Microfluidic Chips

Microfluidic devices were designed in AutoCAD and fabricated by CO_2_ laser micromachining (LES-10, Laser Life Co., Taiwan) on PMMA sheets (50% power, 15% speed). The final chip dimensions were 65 × 35 × 4 mm. The etched plates were ultrasonically cleaned in deionized water for 30 min, washed with ethanol, and baked at 105 °C overnight. After alignment, the PMMA sheets were thermally bonded to glass substrates, and fluidic inlets/outlets were connected using PEEK tubing fixed with AB epoxy adhesive.

### 4.4 Generation of Conductive Microbeads via Microfluidic Emulsification

The aqueous prepolymer phase was composed of 7.5 wt % GelMA, 0.5 wt % poly­(3,4-ethylenedioxythiophene)/poly­(styrenesulfonate) (PEDOT:PSS)­(Clevios PH1000, Heraeus), 400 μg mL^–1^ AuCDs, and 0.5 wt % Irgacure 2529 (Sigma-Aldrich) in 3 mL of deionized water. The oil phase consisted of 5 wt % Span 80 in liquid paraffin. Both phases were introduced into the microfluidic device using syringe pumps at controlled flow rates and observed under an inverted microscope (Eclipse TE2000-U, Nikon). Droplets collected at the outlet were photo-cross-linked by UV irradiation (365 nm, 19 mW cm^–2^, 20 s; OmniCure 1500 series) to form uniform microbeads. The microbeads were sequentially washed three times with hexane and centrifuged in deionized water to remove residual oil and surfactant. After phase separation, the microbeads were collected from the aqueous phase and stored at −4 °C. All procedures were conducted above 20 °C to prevent premature GelMA gelation.

For fluorescence visualization, the GelMA-based microbeads and the 1,2-dioleoyl-3-trimethylammonium-propane chloride (DOTAP)­(Sigma-Aldrich) coating were independently fluorescently labeled prior to surface assembly. The conductive microbeads were first stained with 0.01% (w/v) rhodamine B isothiocyanate (RITC) by incubation at 4 °C overnight to label the hydrogel matrix. After each staining step, unbound dye was removed by three rounds of washing with deionized water. Separately, the DOTAP lipid solution was mixed with 0.01% (w/v) DiD perchlorate before thin-film hydration to fluorescently label the lipid layer. The prelabeled RITC microbeads were then coated with the DiD-labeled DOTAP solution to obtain dual-labeled conductive microbeads, in which the red RITC fluorescence indicated the GelMA core and the far-red DiD fluorescence represented the DOTAP shell. Confocal fluorescence imaging was performed using a laser scanning confocal microscope (ZEISS LSM 800; Carl Zeiss, Oberkochen, Germany) equipped with Plan-Neofluar 20×/0.5 and oil-immersion Fluar 40×/1.30 and 63×/1.40 objectives. The merged fluorescence channels were analyzed to confirm that DOTAP uniformly covered the microbead surface. Microbead diameters were determined from captured images using Nikon Eclipse TE2000-U software.

### 4.5 Surface Functionalization and Assembly of AES

To obtain gene-loading microbeads (AES), positively charged DOTAP-coated microbeads were first prepared. A 10 mg mL^–1^ DOTAP–ethanol stock solution was evaporated to dryness using a thin-film hydration method and subsequently rehydrated in deionized water to yield a stable DOTAP coating solution. Given the difficulty of precisely quantifying the surface area of each microbead, an excess amount of DOTAP solution was incubated with 10 mg of preformed D@PCB under gentle agitation for 30 min to ensure complete surface coverage. The coated microbeads were washed three times with deionized water to remove unbound lipids. Finally, plasmid DNA encoding the miR-6236-targeting sponge was electrostatically adsorbed onto the positively charged DOTAP-modified surface through charge attraction and hydrogen bonding, yielding the final AES.

### 4.6 Characterizations of AuCDs

The morphology and crystalline structure of the AuCDs were examined by transmission electron microscopy (TEM; JEM-2100, JEOL, Japan). For TEM analysis, the nanoparticles were deposited on carbon-coated copper grids and air-dried at room temperature. Digital images were acquired from multiple regions to obtain representative lattice structures, and elemental mapping was performed using an energy-dispersive spectroscopy (EDS) detector (Oxford MAX150) integrated with the TEM. The hydrodynamic diameter and surface charge of the nanoparticles were determined using a Nano-ZS Zetasizer (Malvern Instruments, UK). Samples were dispersed in deionized water and measured in disposable cuvettes; particle size distributions were obtained from the dynamic scattering of light during each measurement cycle. High-resolution X-ray photoelectron spectroscopy (HRXPS; PHI Quantera SXM, Japan) was employed to analyze the surface composition and elemental states of the nanoparticles. Fourier-transform infrared spectroscopy (FT-IR; Horiba F730) was used to identify the characteristic vibrational peaks of GSH ligands and Au–thiolate coordination. X-ray diffraction (XRD; APEX DUO, Bruker) was performed using Cu Kα radiation (λ = 1.5406 Å) over a 2θ range of 10–80° to determine crystallinity and phase composition. Optical absorption was characterized using a UV spectrometer (Tecan Infinite M Plex with NanoQuant plate), while photoluminescence spectra were recorded on a spectrofluorometer (FluoroMax-4, HORIBA Jobin Yvon) to confirm the characteristic orange emission of the AuCDs. Electron paramagnetic resonance (EPR) spectra were recorded using a BRUKER ELEXSYS E-580 spectrometer (Bruker, Germany) to evaluate free–radical interactions of the AuCDs. The peroxidase-like catalytic activity and antioxidant behavior were further assessed using a 3,3′,5,5′-tetramethylbenzidine (TMB) assay, in which 50 μL of Fe_3_O_4_ (3 mg mL^–1^) was mixed with 10 μL of H_2_O_2_ (2.5 mM) and reacted for 5 min, followed by addition of 10 μL of TMB (5 mg mL^–1^) and 930 μL of deionized water. The absorbance change was monitored at 25 °C using UV–Vis spectroscopy to quantify the reaction kinetics.

### 4.7 Rheological Property of MB

The rheological behavior of the conductive microbead hydrogels was evaluated using a rotational rheometer (AR 200ex, TA Instruments, USA) equipped with a 20 mm parallel-plate geometry and a 0.5 mm gap at a controlled temperature of 25 °C. The microbead hydrogel (MBH) scaffolds were placed on the lower plate, and excess sample was carefully removed to ensure uniform contact. Small-amplitude oscillatory shear tests (strain 0.1–10%) were first performed to determine the linear viscoelastic region. Within this range, a constant strain was selected for frequency sweep tests (0.5–5 Hz). Oscillatory stress and strain sweeps were also carried out under identical conditions. The storage modulus (*G*′) and loss modulus (*G*″) were recorded during a time sweep for 5 min at a constant stress of 1 Pa and frequency of 1 Hz to assess viscoelasticity and self-healing behavior.

### 4.8 Electrical Conductivity Test

To evaluate the electrical conductivity of the hydrogels, cylindrical samples (cross-sectional area 3.14 cm^2^, length 1.0 cm) were prepared and placed between two fluorine-doped tin oxide (FTO) glass substrates (1 × 10 cm, Ruilong, Model RLA2K20100) serving as conductive contacts. The conductive surface of each FTO substrate was verified using a digital multimeter before assembly. The hydrogel samples were positioned at the center of the FTO slides and dried overnight at 50 °C to ensure firm adhesion. On the following day, copper wires wrapped with aluminum foil were attached to both ends of the FTO electrodes to establish stable electrical connections. The assembled device was connected to a digital multimeter (Dawson Instruments) to record current–time (*I*–*T*) responses under a constant potential, allowing the evaluation of electrical stability and time-dependent conductivity of the hydrogels.

Electrochemical impedance spectroscopy (EIS) and cyclic voltammetry (CV) were performed using a CHI6116E electrochemical workstation (CH Instruments, Austin, TX, USA) equipped with a screen-printed three-electrode system (DropSens 220AT; Metrohm, Oviedo, Spain) consisting of a gold working electrode, a silver pseudoreference electrode, and a platinum counter electrode. For EIS, measurements were carried out in the frequency range of 0.1 Hz–1 MHz with an AC amplitude of 5 mV and a DC bias of 0.1 V. CV curves were recorded within a potential window of −1.0 to +1.0 V at a scan rate of 0.1 V s^–1^ for 10 consecutive cycles. All measurements were conducted at room temperature under ambient conditions. The obtained impedance spectra were fitted using equivalent circuit models to extract the resistance and capacitive parameters of the conductive microbeads.

To examine local electromechanical responses, piezoresponse force microscopy (PFM) was performed using a scanning probe microscope (Veeco Nanoscope 3100, USA) with conductive Pt/Ir-coated tips. An AC bias of 1 V at 20 kHz was applied to measure amplitude and phase hysteresis loops, revealing the nanoscale polarization behavior and electrical responsiveness of the microbeads.

### 4.9 Degradation of MB

The degradation profile of the microbeads was assessed by monitoring weight loss in phosphate-buffered saline (PBS, pH 7.4) at 37 °C. Briefly, 10 mg of dried microbeads were immersed in a fixed volume of PBS and incubated for predetermined durations (1, 3, 7, 14, 21, 28, and 35 days). At each time point, samples were collected, and the PBS was carefully removed without disturbing the microbeads. The recovered samples were freeze-dried to obtain dry residues, which were then weighed to determine the remaining mass fraction at each interval.

### 4.10 In Vitro Cytotoxicity of Materials

The cytocompatibility of the materials was assessed using the PrestoBlue cell viability assay (Thermo Fisher, Cat. No. A13261). NIH-3T3 fibroblasts were seeded in 96-well plates at a density of 1 × 10^4^ cells per well with 100 μL of complete medium and cultured for 24 h at 37 °C to allow cell attachment. For the indirect extraction test, different particle suspensions were prepared in fresh medium (100 μL), and modified microbeads were immersed in the same medium at 37 °C for 24 h. The resulting supernatants were collected as the extract media. After replacing the culture medium with 100 μL of the corresponding extracts, the cells were further incubated for 24 h. Subsequently, 20 μL of PrestoBlue reagent was added to each well and reacted for 10 min at 37 °C. The absorbance at 570 nm was recorded using a microplate reader (Synergy HT, BioTek Instruments, USA). Cell viability was calculated by normalizing the absorbance of treated samples to that of untreated controls.

### 4.11 In Vitro Coculture of Cells with Conductive D@PCB

To investigate cell–material interactions, NIH-3T3 cells were cultured in 3 cm confocal dishes at a density of 1 × 10^4^ cells mL^–1^ and maintained at 37 °C. RITC-stained D@PCB (100 μL; emission 580 nm) were added to the culture medium and gently agitated to ensure uniform dispersion among the cells. After 7 days of coculture, the cells were fixed with 4% paraformaldehyde, and nuclei were counterstained with DAPI (4′,6-diamidino-2-phenylindole; emission 470 nm). F-actin filaments were visualized using Alexa Fluor 488-phalloidin (emission 510 nm). Excess dye was removed by three PBS washes, and samples were mounted with Fluoromount aqueous mounting medium (Sigma-Aldrich). Imaging was performed on a laser scanning confocal microscope (ZEISS LSM 800; Carl Zeiss, Oberkochen, Germany) equipped with Plan-Neofluar 20×/0.5 and oil-immersion Fluar 40×/1.30 and 63×/1.40 objectives. Confocal *z*-stacks were analyzed to examine microbead localization relative to the nuclei and cytoskeleton, highlighting both two-dimensional adhesion and three-dimensional network formation.

### 4.12 Endosomal and Lysosomal Escape Assay

To evaluate endosomal and lysosomal escape of nanoparticles, two fluorescence colocalization assays were conducted. For endosomal labeling, NIH-3T3 cells were seeded in 3 cm confocal dishes at a density of 5 × 10^5^ cells mL^–1^ and cultured for 24 h. CellLight Early Endosomes-GFP (Invitrogen, Cat. No. C10586) was diluted 1:20 in fresh medium and added to the cultures to transduce GFP-labeled endosomes for 24 h. Subsequently, nanoparticles were introduced and incubated with the cells for 1 h at 37 °C. The medium was then replaced with PBS, and nuclei were stained with Hoechst 33342 (Invitrogen, Cat. No. H3570; 1:1000 in PBS) for 1 h.

For lysosomal visualization, a parallel assay was performed using LysoTracker Red (Invitrogen, Cat. No. L7526) together with Hoechst 33342 (1:1000 in PBS). NIH-3T3 cells were precultured for 24 h, followed by nanoparticle exposure for 2 h. The dye mixture was added and incubated for 1 h at 37 °C. After staining, all samples were washed three times with PBS and immediately imaged under a laser scanning confocal microscope (ZEISS LSM 800) maintained at 37 °C. Fluorescence overlap between nanoparticles and endosome/lysosome signals was analyzed to assess intracellular trafficking and endosomal escape efficiency.

### 4.13 In Vitro Differentiation of Neural Stem Cells

For neural stem cell (NSC) culture, confocal dishes were precoated with poly-l-lysine (PLL) to enhance cell adhesion. The dish surface was first activated by oxygen plasma treatment for 2 min, followed by washing three times with 1 mL of Tris buffer (pH 7.4) for 3 min each. Subsequently, 1 mL of PLL solution was added and incubated for 30 min at room temperature. The PLL solution was then removed, and the dishes were again rinsed three times with Tris buffer. After a final addition of 2 mL of Tris buffer, the coated dishes were placed under UV light in a laminar flow hood overnight for sterilization. Before cell seeding, the dishes were washed three times with deionized water. Approximately 200 NSC spheroids were plated per 3 cm confocal dish using a 24-well plate format. After an 8 h attachment period, various materials were introduced for coculture experiments. The differentiation medium consisted of DMEM/F-12 supplemented with 1% penicillin–streptomycin, 1% N-2 supplement, and 0.2% B-27 supplement, differing from the standard maintenance medium. NSC cultures were divided into five experimental groups: (1) control, (2) MB, (3) PCB, (4) D@PCB, and (5) D@PCB + HFMF. For the HFMF group, a high-frequency magnetic field (HFMF; Power Cube 32/900, Honor Industries) was applied at 1 MHz and 2.24 kW for 3 min per day over 7 days.

After the 7-day differentiation period, the cells were rinsed three times with PBS and fixed in 3.7% formaldehyde for 10 min. Fixed samples were then washed three times with PBS and incubated in 5 wt % bovine serum albumin (BSA) blocking buffer for 1 h at room temperature. The blocking solution was replaced with primary antibodies, including rabbit anti-GFAP (1:800) and mouse anti-βIII-tubulin (Tuj1, 1:800), and samples were incubated overnight at 4 °C. After three PBS washes, secondary antibodies—donkey antirabbit IgG (1:800) and goat antimouse IgG-FITC (1:800)—were applied for 2 h at room temperature. Following a final PBS wash, nuclei were counterstained with DAPI mounting medium. Fluorescence imaging was performed using a laser scanning confocal microscope (ZEISS LSM 800; Carl Zeiss, Oberkochen, Germany). For quantitative analysis, fluorescence images were processed with ImageJ software to determine the ratios of GFAP-positive astrocytes and Tuj1-positive neurons outside the neurosphere regions. The differentiation percentages were calculated from the mean intensity of marker-positive cells in each representative field.

### 4.14 In Vitro Gene Functional Assay

All animal experiments were performed following the protocols evaluated and approved by the Institutional Animal Care and Use Committee (IACUC) of National Tsing Hua University (Approval No. 112006). All participants gave informed consent. Seven-week-old female C57BL/6 mice were randomly divided into five experimental groups: (1) control, (2) PCB, (3) D@PCB, (4) AES, and (5) AES + HFMF. Each conductive microbead scaffold was prepared by suspending 1 mg of particles in 20 μL of sterile PBS.

A traumatic brain injury (TBI) model was established by drilling a craniotomy above the left motor cortex (M1/M2 region) and generating a cortical lesion using a 2 mm diameter biopsy punch to a depth of 1.5 mm. The microbead scaffolds were implanted into the lesion cavity 1 day after injury to avoid acute edema. For groups receiving electric stimulation, a high-frequency magnetic field (HFMF; Power Cube 32/900, Honor Industries) was applied at 1 MHz and 2.24 kW for 3 min per day over 14 consecutive days.

## Supplementary Material



## Notes

Elemental mapping of AuCDs (Figure S1); XPS survey spectrum of AuCDs (Figure S2); optical appearance of AuCDs under daylight and UV illumination (Figure S3); antioxidant properties of AuCDs (Figure S4); elemental mapping and morphology of MB and D@PCB obtained by SEM-EDS (Figure S5); schematic illustration of the ethanol-based DOTAP coating process (Figure S6); surface charge and degradation behavior of microbeads (Figure S7); electrochemical and electromechanical properties of microbead (Figure S8); cytotoxicity of individual material components toward NIH-3T3 fibroblasts (Figure S9); real-time thermal monitoring of the AES under continuous HFMF irradiation (Figure S10); cellular uptake of D@PCB by NIH-3T3 fibroblasts (Figure S11); line-profile and 2.5D visualization of endosomal and lysosomal association of D@PCB with and without HFMF (Figure S12); in vitro gene transfection with GFP-encoding plasmids (Figure S13); transfection of NSCs with miR-6236 sponge plasmids (Figure S14); proposed mechanistic model of sponge-mediated miR-6236 suppression in injured neuronal microenvironments (Figure S15); in vivo GFP plasmid transfection in a mouse TBI model (Figure S16); in vivo expression of the miR-6236 sponge dual-reporter plasmid following microbead implantation (Figure S17); long-term in vivo neurovascular regeneration and glial microenvironment modulation (Figure S18); long-term assessment of inflammatory polarization markers (Figure S19); DTI-based fiber tractography of distinct sensorimotor pathways and lesion-centered tracking (Figure S20); in vivo long-term systemic biosafety evaluation of the AES platform (Figure S21) (PDF)
