# Supplementary material for: Wireless Electromagnetic Generation of miRNA Sponges and Nerve Stimulation by an Adaptable Electrical Scaffold for Repair of Traumatic Brain Injury
Source: ACS Nano. 2026 May 25;20(22):16365–86. doi: 10.1021/acsnano.6c04759 (PMC13255529; doi:10.1021/acsnano.6c04759)
Supplement: Supplementary file 1 [file nn6c04759_si_001.pdf]

Supplementary Information

Wireless Electromagnetic Generation of miRNA Sponges and Nerve Stimulation by An Adoptable Electrical Scaffold for Repair of Traumatic Brain Injury

Hoi Man Iao[1], Wan-Chi Pan[1], Yun-Hsuan Chang[1], Ngoc-Tri Tran[1], Hsiu-Ching Liu[1], Ru-Siou Hsu[2], Tsu-Chin Chou[3], I-Chi Lee[1], Lun-De Liao[4],Wen-Hsuan Chiang[5], Ssu-Ju Li[2], Eric Hwang[6,7,8], Ming-You Shie[10,11,12], You-Yin Chen[2,*], Shang-Hsiu Hu[1,3,9*]

[1] Department of Biomedical Engineering and Environmental Sciences, National Tsing Hua University, Hsinchu 300044, Taiwan

[2] Department of Biomedical Engineering, National Yang Ming Chiao Tung University, Taipei, 112304 Taiwan

[3] Institute of Analytical and Environmental Sciences, National Tsing Hua University, Hsinchu, 300044 Taiwan

[4] Institute of Biomedical Engineering and Nanomedicine, National Health Research Institutes, Miaoli County, 35053 Taiwan

[5] Department of Chemical Engineering, National Chung Hsing University, Taichung 402, Taiwan

[6] Institute of Molecular Medicine and Bioengineering, National Yang Ming Chiao Tung University, Hsinchu, 300093 Taiwan

[7] Department of Biological Science and Technology, National Yang Ming Chiao Tung University, Hsinchu, 300093 Taiwan

[8] Center for Intelligent Drug Systems and Smart Bio-devices (IDS2B), National Yang Ming Chiao Tung University, Hsinchu, 300093 Taiwan

[9] Institute of Biomedical Engineering, National Tsing Hua University, Hsinchu, 300044 Taiwan

[10] Department of Biomedical Engineering, China Medical University, Taichung, 406040, Taiwan

[11] Research & Development Center for x-Dimensional Extracellular Vesicles, China Medical University Hospital, Taichung, 404332, Taiwan

[12] Xenotransplantation Translational Research Center, China Medical University Hospital, Taichung, 404332, Taiwan

E-mail addresses: youyin.chen@nycu.edu.tw; shhu@mx.nthu.edu.tw

Methods

In vitro gene functional assay

Gene functional assays were performed to evaluate plasmid transfection efficiency and the biological effects of miR6236 sponge constructs. For preliminary evaluation, NIH-3T3 fibroblasts were transfected with EGFP-expressing plasmids to verify delivery performance and fluorescence expression. For functional assessment in neural

stem cells (NSCs), glass coverslips placed in 24-well plates were pre-coated with 150 μL of poly-L-lysine (PLL) overnight at room temperature to promote cell adhesion. The following day, coverslips were rinsed three times with sterile double-distilled water (ddH_2O). Approximately 200 NSC spheroids were seeded per well and allowed to attach firmly to the coated surface. After four days of culture, microbeads containing plasmid DNA—either miR6236 sponge or control sponge constructs—were added to each well and co-incubated with NSCs for 72 h. Fixation and immunofluorescence staining were carried out following the same protocol as described in the NSC differentiation analysis section. Confocal imaging (ZEISS LSM 800; Carl Zeiss, Oberkochen, Germany) was used to visualize EGFP expression and evaluate gene modulation effects within the neural spheroids.

Brain collection and immunofluorescence staining

At the designated endpoints, mice were euthanized, and whole brains were collected and fixed in 4% paraformaldehyde (PFA) for 12h at 4 °C. Fixed tissues were cryoprotected by sequential immersion in 10% sucrose (30 min), 20% sucrose (overnight), and 30% sucrose (overnight) solutions. The brains were then embedded in optimal cutting temperature (OCT) compound and cryosectioned into 10-μm slices at −20 °C. Sections were post-fixed in 100% methanol at −20 °C for 5 min and washed three times with PBS.

To block nonspecific antibody binding, the sections were incubated for 1 h at room temperature in PBS containing 5% bovine serum albumin (BSA), 0.1% Tween-20, and 0.25% Triton X-100. Primary antibodies were applied overnight at 4 °C, including rabbit anti-GFAP (1:800, Abcam) for astrocytes, rabbit anti-NF200 (1:800, Abcam) for neurofilament regeneration, goat anti-Iba1 (1:800, Abcam) for microglia, and rat anti-CD31 (1:800, Abcam) for endothelial cells. After washing three times with PBS, the sections were incubated with secondary antibodies for 2 h at room temperature: donkey anti-rabbit IgG-Alexa Fluor 647 (1:800), rabbit anti-goat IgG-Alexa Fluor 488 (1:800), and goat anti-rat IgG-Alexa Fluor 488 (1:800). Finally, the samples were rinsed with PBS and counterstained with DAPI before mounting.

Fluorescence imaging was carried out using a laser scanning confocal microscope (ZEISS LSM 800; Carl Zeiss, Oberkochen, Germany). Quantitative analysis of fluorescence intensity and the proportion of marker-positive cells was performed using ImageJ software to evaluate glial activation, neuronal regeneration, and vascular remodeling in the peri-lesion regions.

Animal behavior assessment

To evaluate motor coordination and limb function recovery after traumatic brain injury, three behavioral tests were conducted: the cylinder test, the grid-walking test, and the pasta-handling test. Behavioral performance was assessed on days −1, 3, 7, 14, 21, and 28 post-injury.

In the cylinder test, forelimb usage asymmetry was analyzed to assess sensorimotor integration. Each mouse was placed individually in a transparent Plexiglas cylinder (height 15 cm, diameter 10 cm), and forelimb wall contacts during vertical exploration were recorded. The number of left (L) and right (R) forelimb contacts was counted, and the asymmetry index was calculated using the following expression:

$$\text{Asymmetry index} = \frac{[R - L]}{[R + L]}.$$

A higher value indicated greater unilateral motor impairment. The grid-walking test was used to evaluate hindlimb coordination. Mice were placed on a horizontal metal grid (grid size 2.5 × 2.5 cm^2, test area 12 × 36 × 10 cm), and the number of foot-faults—instances when a paw slipped through the openings—was recorded. The error rate was expressed as:

$$\text{Foot-fault rate} = \frac{\text{number of foot-faults}}{\text{total steps}} \times 100\%.$$

The pasta-handling test was designed to assess fine motor control of the forelimbs. Uncooked pasta strands (1 mm diameter, 2.6 cm length) were provided to each mouse, and the time required to completely consume a single piece was recorded as a measure of forelimb dexterity.

Diffusion MRI after treatments

All diffusion MRI (dMRI) experiments were performed in compliance with institutional ethical guidelines for animal research. Imaging was conducted on a 7 T small-animal MRI scanner (BioSpec 70/30 USR, Bruker, Ettlingen, Germany) equipped with a 30 cm bore. Radiofrequency excitation was delivered via a linear volume coil, and signals were received using a planar surface coil (T7399V3; Bruker, Billerica, MA, USA) positioned over the mouse head. Anesthesia was induced with 3% isoflurane (Attane™, Minrad Inc., NY, USA) in a mixed gas flow of 20% O_2, 75% N_2,

and 5% CO_2. Throughout scanning, animals were immobilized in a custom holder, and body temperature was maintained at 37 °C using a feedback-controlled heating system. Respiratory rate was continuously monitored (SA Instruments, Inc., New York, USA) and maintained at 65–75 breaths min^{-1}.

Magnetic field homogeneity was optimized using the FASTMAP shimming routine over a 7 × 7 × 7 mm^3 isotropic voxel encompassing the imaging region. Diffusion-weighted images were acquired using a double-shot spin-echo echo-planar imaging (EPI) sequence (15 axial slices, 0.4 mm thickness, field of view = 20 × 20 mm^2, matrix = 80 × 80, TR = 3,750 ms, TE = 31.18 ms, bandwidth = 750 kHz). Data were collected along 30 diffusion directions with b values of 0, 1,000, and 2,000 s mm^{-2}.

Diffusion tensor imaging (DTI) datasets were processed in DSI Studio (http://dsi-studio.labsolver.org/). Pre-processing included motion correction, brain extraction, and registration to the first b_0 image. Fiber tractography was reconstructed using the DTI algorithm, and tract-based findings were subsequently correlated with histological and behavioral outcomes to evaluate post-treatment neural network recovery.

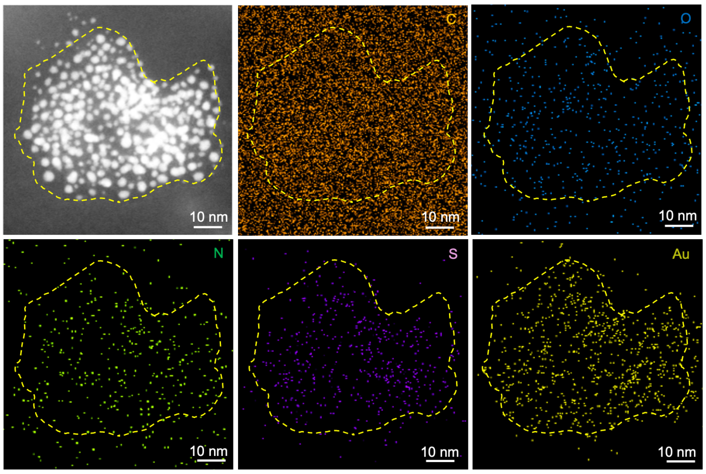

Figure S1. Elemental mapping of AuCDs. EDS elemental mapping images of AuCDs show the homogeneous spatial distribution of C, O, N, S, and Au within each nanodot, confirming uniform incorporation of Au and heteroatoms in the GSH-derived carbon framework.

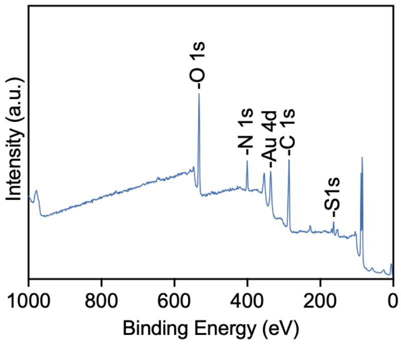

Element	Atomic (%)
C	54.5
O	28.5
N	10.0
S	3.6
Au	3.4

Figure S2. XPS survey spectrum of AuCDs. The full-range XPS survey spectrum reveals the coexistence of C 1s, N 1s, O 1s, S 2p, and Au 4f signals, confirming the incorporation of Au species within the GSH-derived carbon matrix.

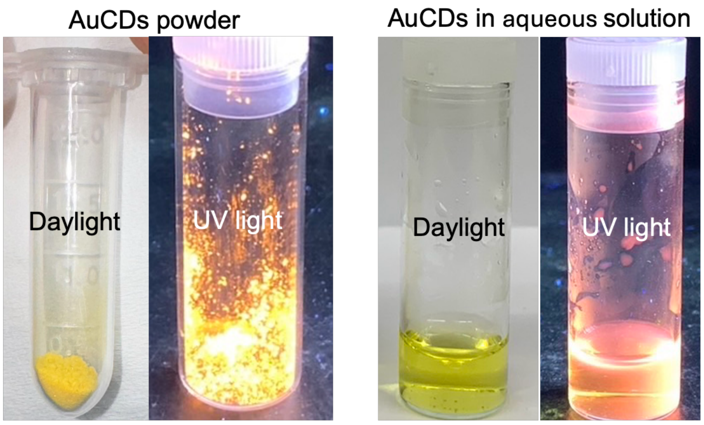

Figure S3. Optical appearance of AuCDs under daylight and UV illumination. Photographs of AuCDs in powder and aqueous solution under daylight and 365 nm UV light show bright orange photoluminescence, confirming their excitation-dependent emission and colloidal dispersibility.

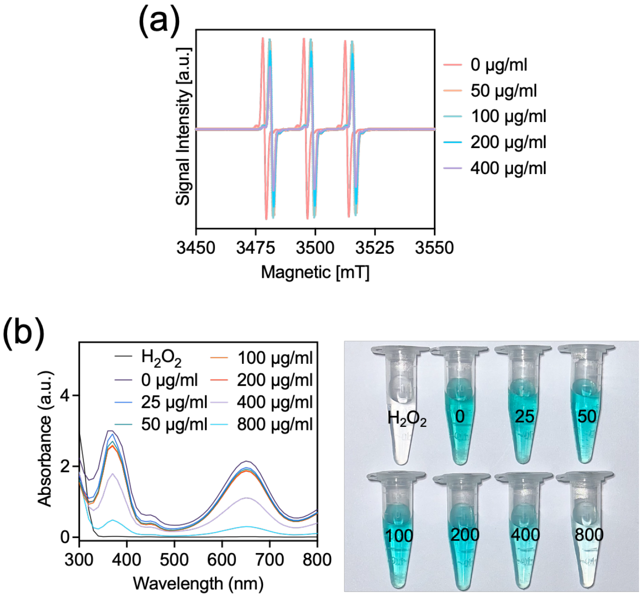

Figure S4. Antioxidant properties of AuCDs. (a) Electron paramagnetic resonance (EPR) spectra obtained using TEMP as a spin probe confirm efficient $^1O_2$ quenching by AuCDs. (b) 3,3,5,5-Tetramethylbenzidine (TMB) colorimetric assay showing concentration-dependent ROS scavenging activity, demonstrating their redox-interactive surface states.

(a)

(b)

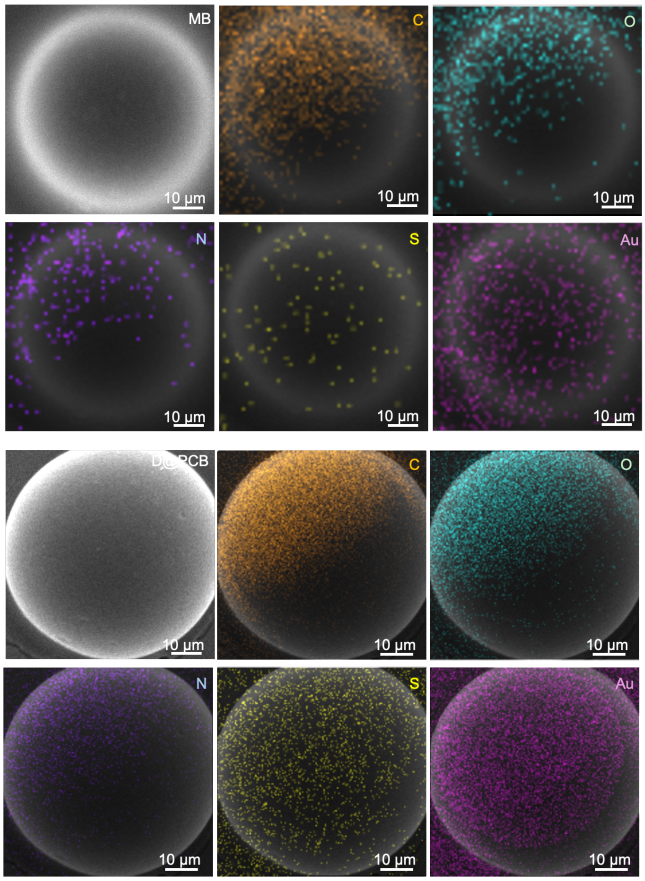

Figure S5. Elemental mapping and morphology of MB and D@PCB obtained by SEM-EDS. (a) Scanning electron microscopy (SEM) and corresponding energy-dispersive X-ray spectroscopy (EDS) elemental maps of GelMA MBs showing smooth spherical morphology with detectable signals of carbon (C), oxygen (O) and nitrogen (N). The sulfur (S) and gold (Au) channels are shown for reference and did not exhibit discernible signal. (b) SEM–EDS mapping of D@PCB displaying uniform morphology and widespread distribution of C, O, N, S and Au signals across the particle section.

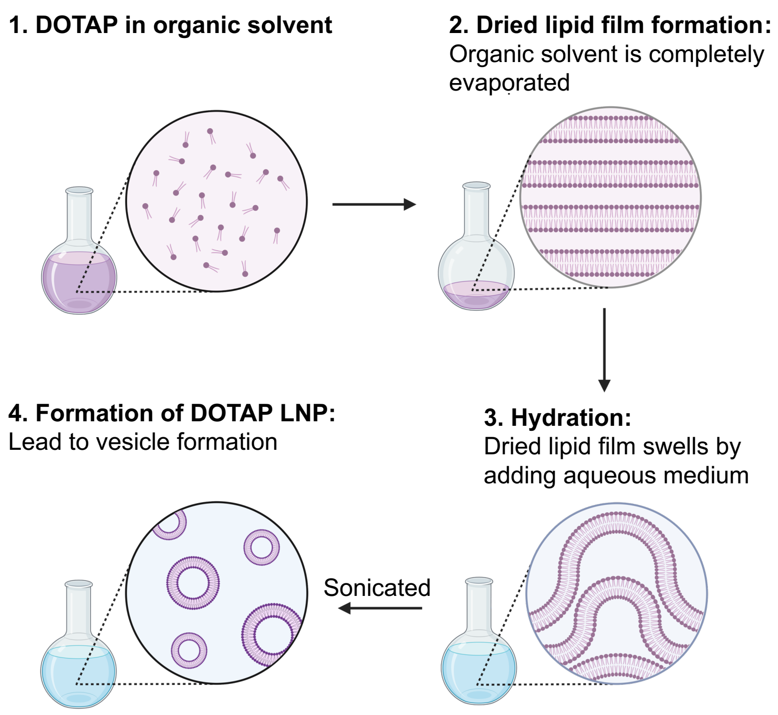

Figure S6. Schematic illustration of the ethanol-based DOTAP coating process. DOTAP lipids were first dissolved in absolute ethanol and deposited to form a uniform thin lipid film upon solvent evaporation. During subsequent rehydration in aqueous medium, the dried film reorganized into multilamellar vesicle (MLV) structures through spontaneous self-assembly of the cationic amphiphiles. Mild sonication disrupted the stacked bilayers and promoted the transition from MLVs to small unilamellar vesicle (SUV)-like assemblies. These nanoscale vesicles exhibit higher curvature and greater exposure of positively charged headgroups, facilitating electrostatic association with the negatively charged hydrogel surface of the PEDOT:PSS–GelMA microbeads. This process yields a uniform cationic DOTAP coating that enhances nucleic acid adsorption and transfection efficiency while maintaining structural simplicity compared to conventional liposomal formulations.

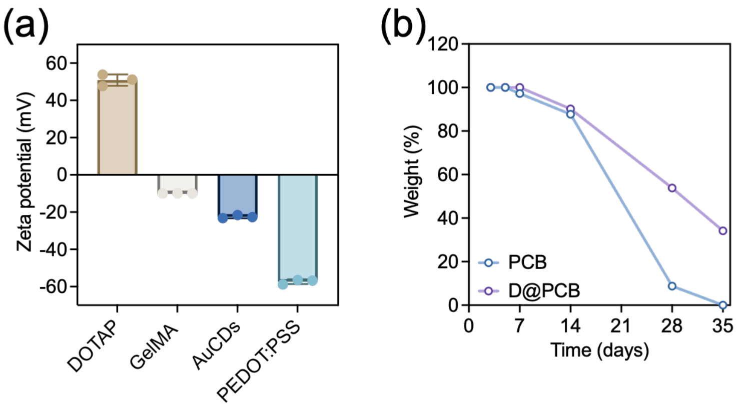

Figure S7. Surface charge and degradation behavior of microbeads. (a) Zeta-potential measurements of the main components used for microbead fabrication, including GelMA, PEDOT:PSS, AuCDs and DOTAP. (b) Degradation of PCB and D@PCB in phosphate-buffered saline (PBS, 37 °C) over 35 days.

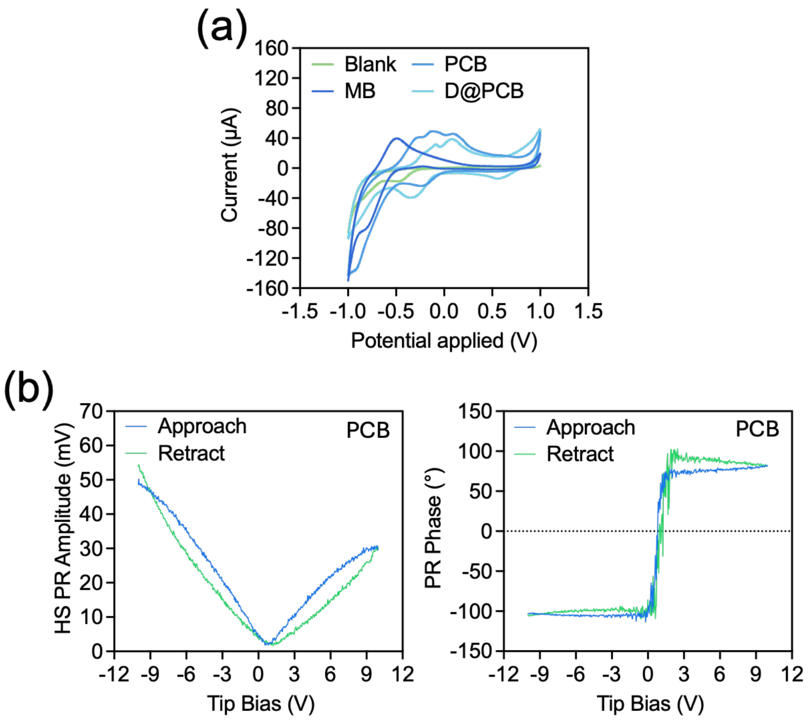

Figure S8. Electrochemical and electromechanical properties of microbead. (a) Cyclic-voltammetry (CV) curves of MB, PCB and D@PCB gels measured at identical scan rates, showing reproducible redox responses. (b) High-sensitivity piezoresponse (HS-PR) amplitude and phase mappings collected from PCB, revealing detectable local electromechanical signals under the applied alternating bias.

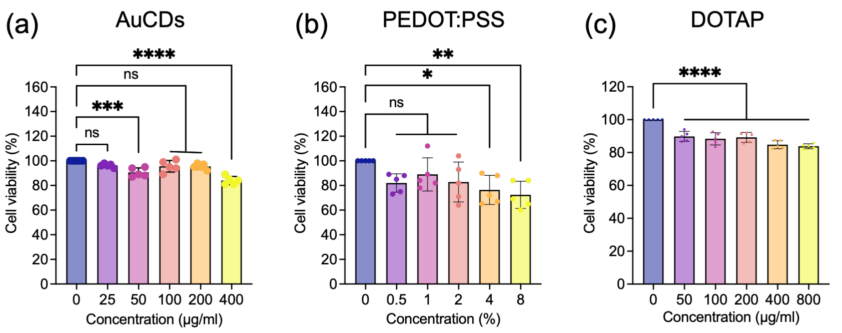

Figure S9. Cytotoxicity of individual material components toward NIH-3T3 fibroblasts. Cell viability was evaluated after 24 h incubation with increasing concentrations of (a) AuCDs, (b) PEDOT:PSS, and (c) DOTAP using the PrestoBlue assay. Data are expressed as mean ± s.d. (n = 5). All materials exhibited high cell viability (> 80 %) across the tested concentration range, suggesting negligible acute cytotoxicity under these in-vitro conditions.

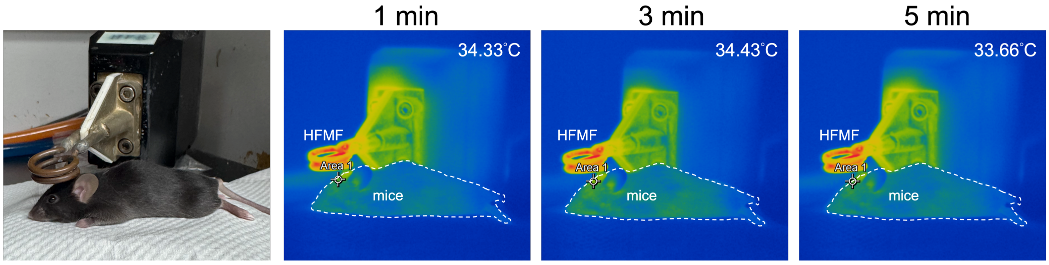

Figure S10. Real-time thermal monitoring of the AES under continuous HFMF irradiation (1 MHz, 2.24 kW). Infrared thermal images captured at 1, 3, and 5 minutes demonstrate that the macroscopic temperature remains below 34°C, well below physiological body temperature, ensuring exceptional thermal safety during the standard 3-minute therapeutic window.

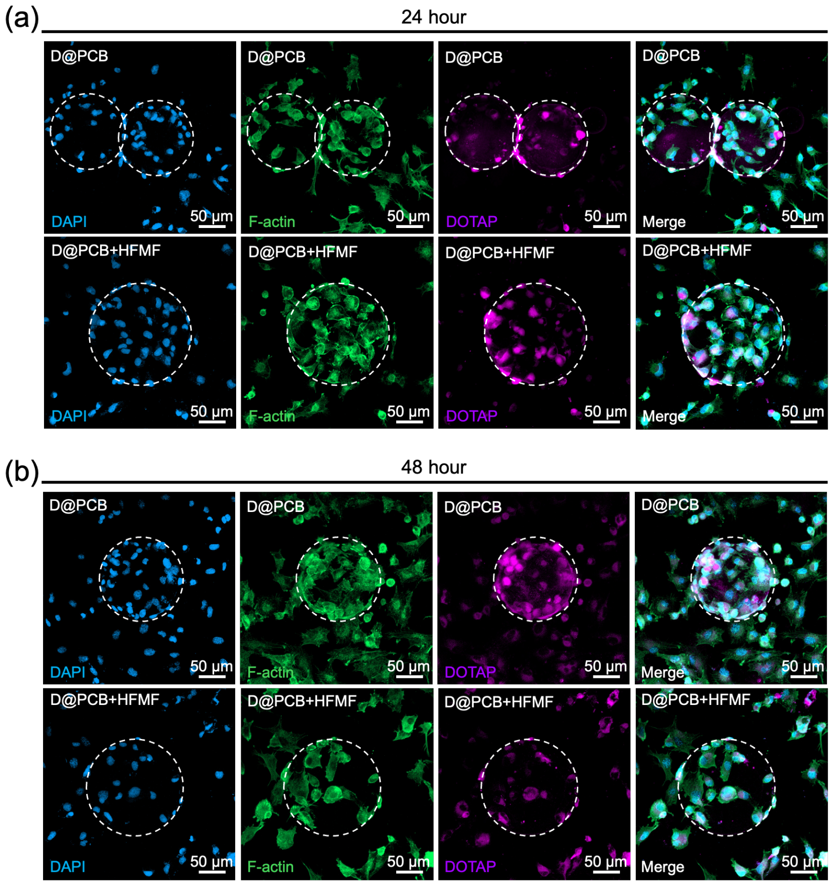

Figure S11. Cellular uptake of D@PCB by NIH-3T3 fibroblasts. (a) CLSM images of NIH-3T3 cells co-cultured with D@PCB for 24 h. (b) CLSM images of cells after 48 h of co-culture. Nuclei were stained with DAPI (blue) and F-actin with Alexa Fluor 488 phalloidin (green). The microbead positions are indicated by dashed circles.

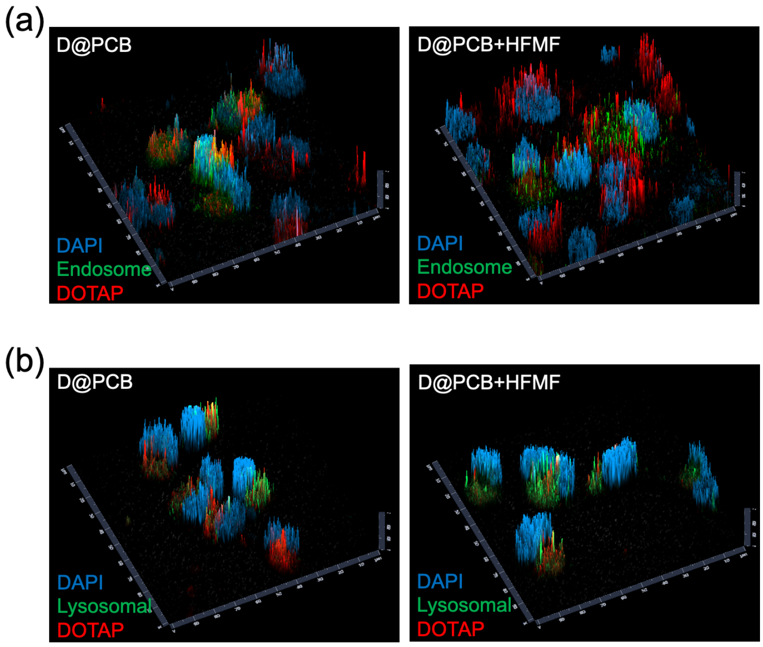

Figure S12. Line-profile and 2.5D visualization of endosomal and lysosomal association of D@PCB with and without HFMF. (a) CLSM 2.5D reconstruction images of NIH-3T3 cells after incubation with D@PCB or D@PCB under HFMF stimulation, showing the spatial relationship between endosomes (green) and DOTAP fluorescence (red) within DAPI-stained nuclei (blue). (b) 2.5D visualization of lysosomal staining under identical conditions. The plots display fluorescence intensity profiles along the z-axis for each channel, illustrating relative signal overlap in cells treated with or without HFMF. The spatial relationship between lysosomal (green) and DOTAP fluorescence (red) within DAPI-stained nuclei (blue).

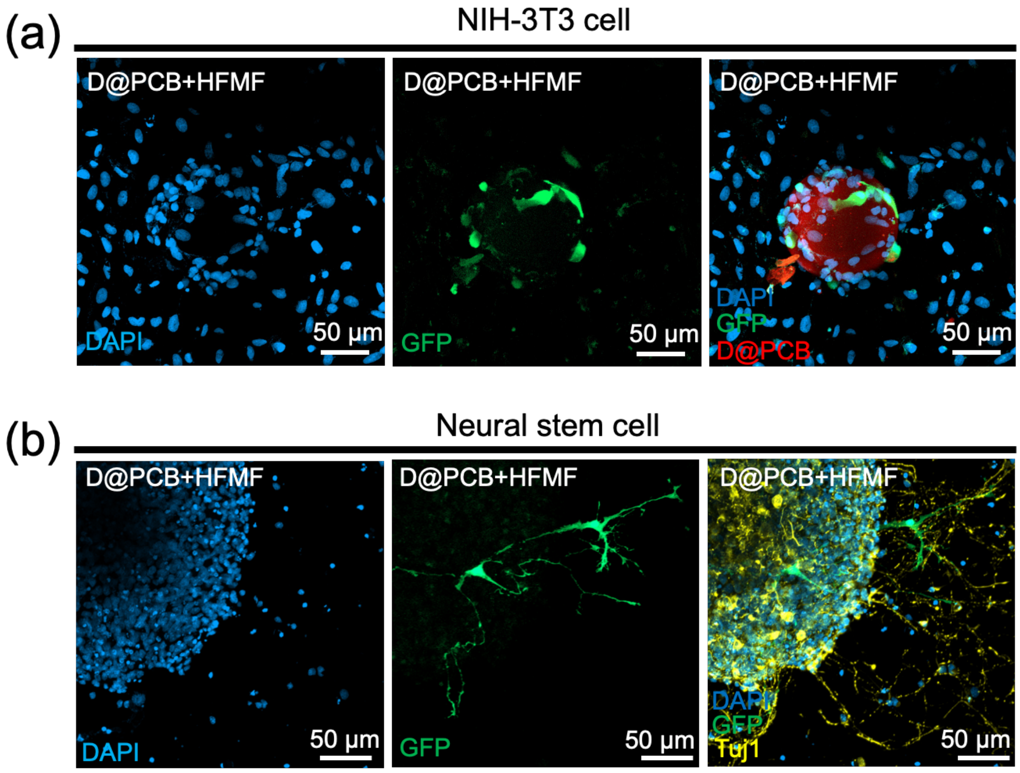

Figure S13. *In vitro* gene transfection with GFP-encoding plasmids. (a) CLSM images of NIH-3T3 cells co-cultured with D@PCB under HFMF stimulation; microbeads are visible in the images. GFP signals (green) were detected in cells surrounding the microbeads. (b) Confocal images of neural stem cells (NSCs) exposed to the same transfection conditions. GFP fluorescence was observed in cells within and around the neurospheres, while the NSC spheroids remained compact. Nuclei were stained with DAPI (blue).

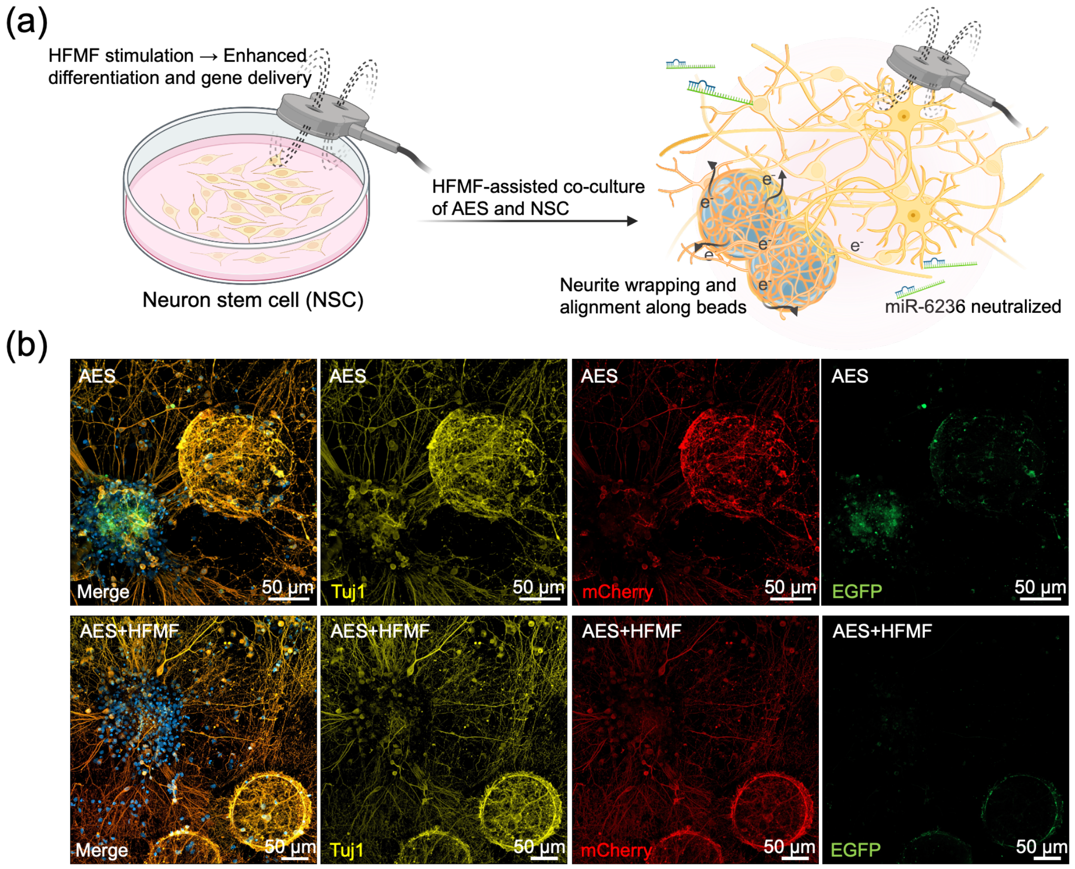

Figure S14. Transfection of NSCs with miR-6236 sponge plasmids. (a) Schematic illustration of *in vitro* co-culture of NSCs with miR-6236 sponge-loaded conductive microbeads (AES). Upon exposure to HFMF stimulation, localized electric cues generates electrical microcurrents that facilitate plasmid uptake and enhance neuronal differentiation. (b) Representative CLSM images of NSC cultures treated with miR-6236 sponge-loaded D@PCB. Neurite networks extended radially from neurospheres and were found in close proximity to the microbeads. Samples exposed to HFMF showed denser neurite arrangements and broader areas of Tuj1-positive (yellow) extensions surrounding the microbeads. Red (mCherry) and green (EGFP) fluorescence correspond to the two reporter sequences encoded in the miR-6236 sponge construct, and nuclei were counterstained with DAPI (blue).

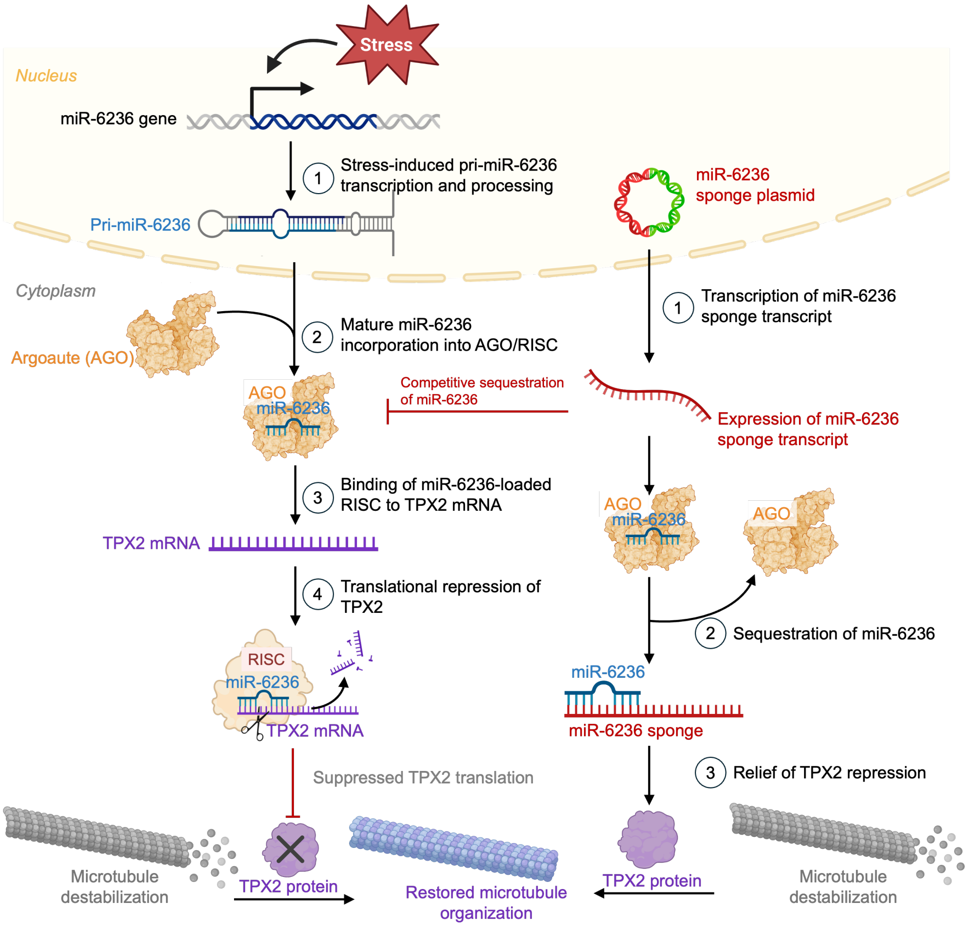

Figure S15. Proposed mechanistic model of sponge-mediated miR-6236 suppression in injured neuronal microenvironments. Stress-associated induction of miR-6236 promotes its biogenesis and loading into the AGO/RISC complex, which is proposed to repress pro-regenerative cytoskeletal programs, illustrated here through a TPX2-associated microtubule regulatory axis. This repression is associated with reduced TPX2 translation, microtubule disorganization, and impaired neurite extension. In contrast, AES-mediated delivery and expression of the miR-6236 sponge transcript competitively sequesters miR-6236, thereby relieving this repression and restoring microtubule organization. The resulting cytoskeletal stabilization is consistent with the enhanced neurite outgrowth and improved neuronal interconnection observed after miR-6236 depletion. This schematic is intended as a mechanistic working model integrating prior literature and the present functional observations.

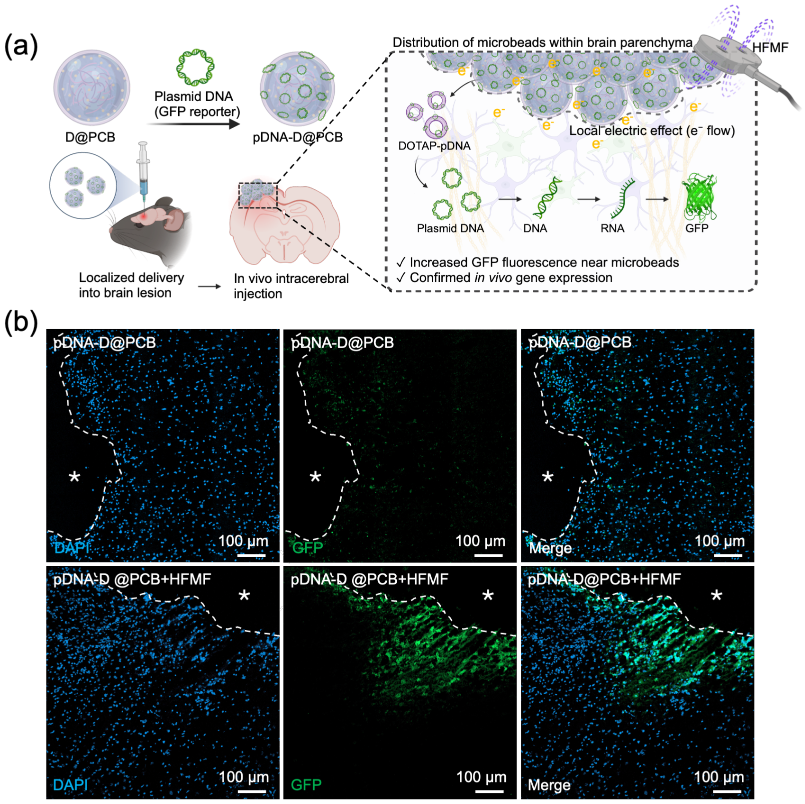

Figure S16. *In vivo* GFP plasmid transfection in a mouse TBI model. (a) Schematic illustration of *in vivo* gene delivery using DOTAP-coated conductive microbeads (D@PCB). The system enables plasmid DNA (pDNA) encapsulation within hydrogel microbeads and facilitates electric-assisted transfection under HFMF stimulation. (b) Representative CLSM images of brain sections collected seven days after implantation. Distinct GFP expression (green) was observed near the lesion cavity in animals treated with pDNA-D@PCB, confirming successful *in vivo* gene transfection. In the presence of HFMF stimulation, GFP fluorescence became more continuous and intense along the lesion boundary, indicating enhanced transfection efficiency. Asterisks mark the cavity margin; nuclei are counterstained with DAPI (blue).

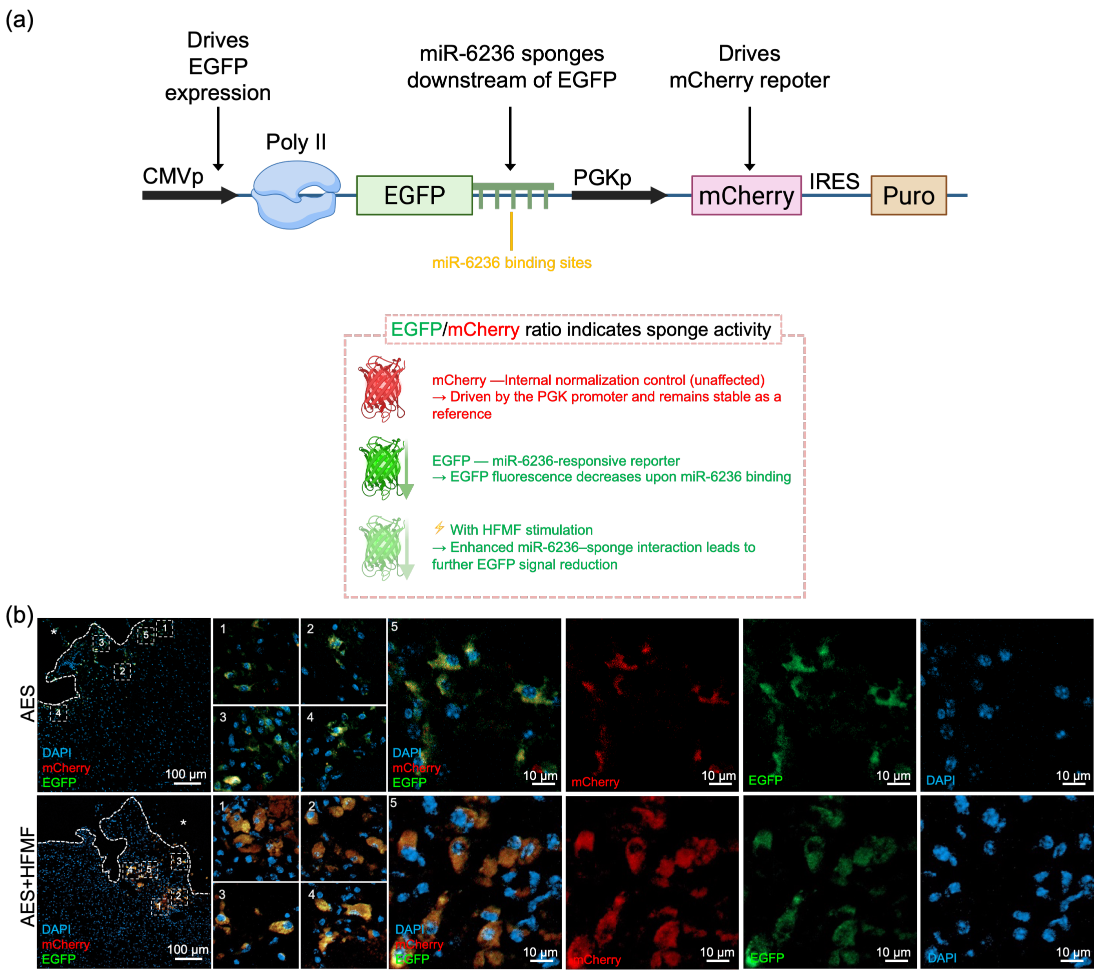

Figure S17. *In vivo* expression of the miR-6236 sponge dual-reporter plasmid following microbead implantation. (a) Schematic illustration of the dual-reporter miR-6236 sponge construct, in which EGFP (green) serves as the miR-6236-responsive reporter containing complementary binding sites in its 3'UTR, while mCherry (red) functions as an internal normalization control driven by an independent promoter. Under HFMF stimulation, enhanced miR-6236 sponge interactions lead to further suppression of EGFP fluorescence. (b) Representative confocal images of brain sections collected from mice implanted with AES, with or without HFMF stimulation. High-magnification views of peri-lesional regions show cells co-expressing mCherry and EGFP, as well as cells predominantly exhibiting mCherry fluorescence, reflecting differential sponge activity depending on electric stimulation. mCherry (red) and EGFP (green) indicate expression of the dual-reporter construct, while DAPI (blue) marks cell nuclei. Asterisks mark the cavity margin.

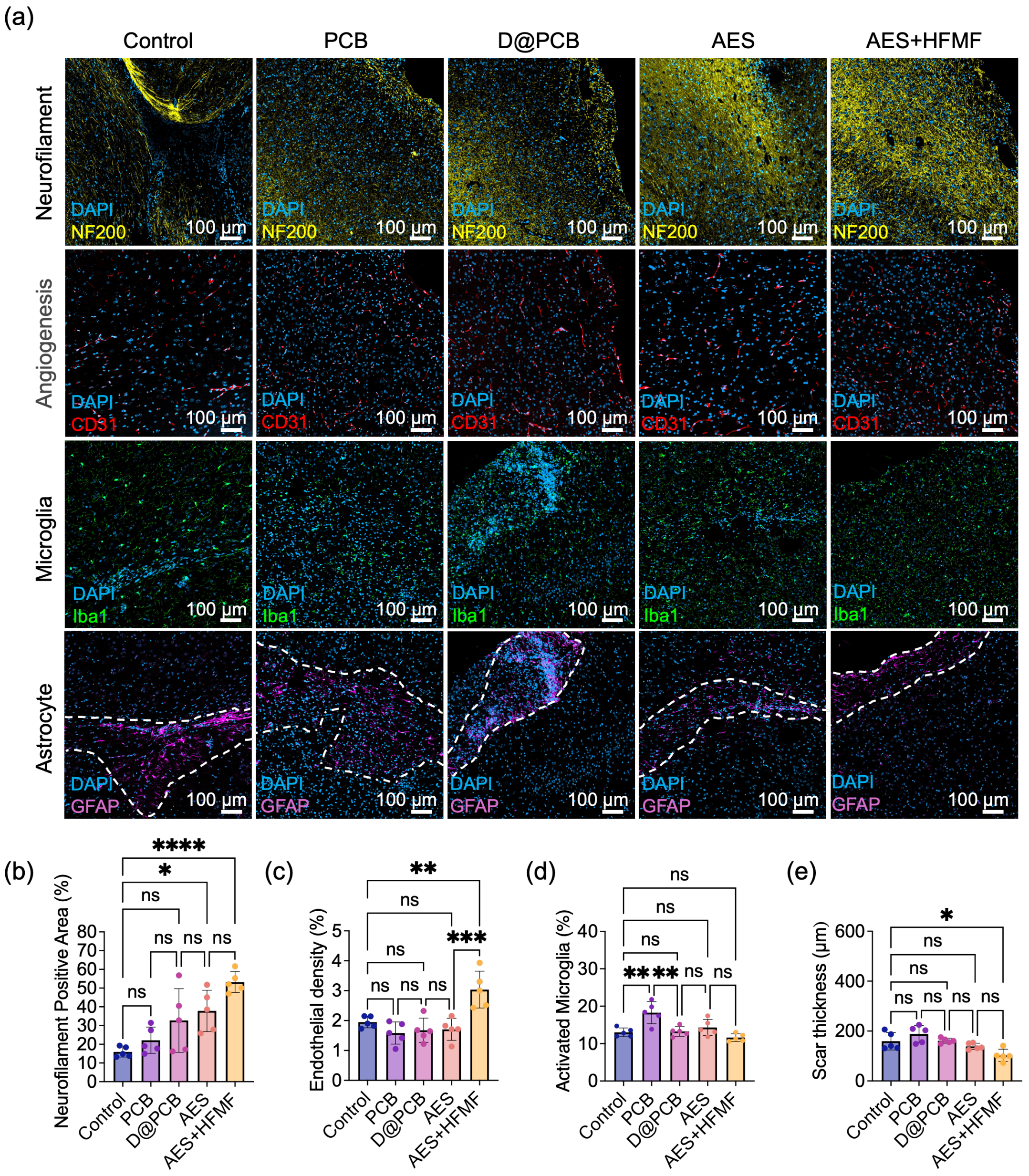

Figure S18. Long-term *in vivo* neurovascular regeneration and glial microenvironment modulation at 67 days post-TBI. (a) Peri-lesional immunostaining at day 67 showing neurons (NF200, yellow), endothelial cells (CD31, red), microglia/macrophages (Iba1, green) and astrocytes (GFAP, purple), with DAPI (blue) marking nuclei. (b-e) Quantitative analyses of NF200^{+} area, endothelial density, activated microglia and glial-scar thickness (n = 5, mean ± s.d.; one-way ANOVA with Tukey's test).

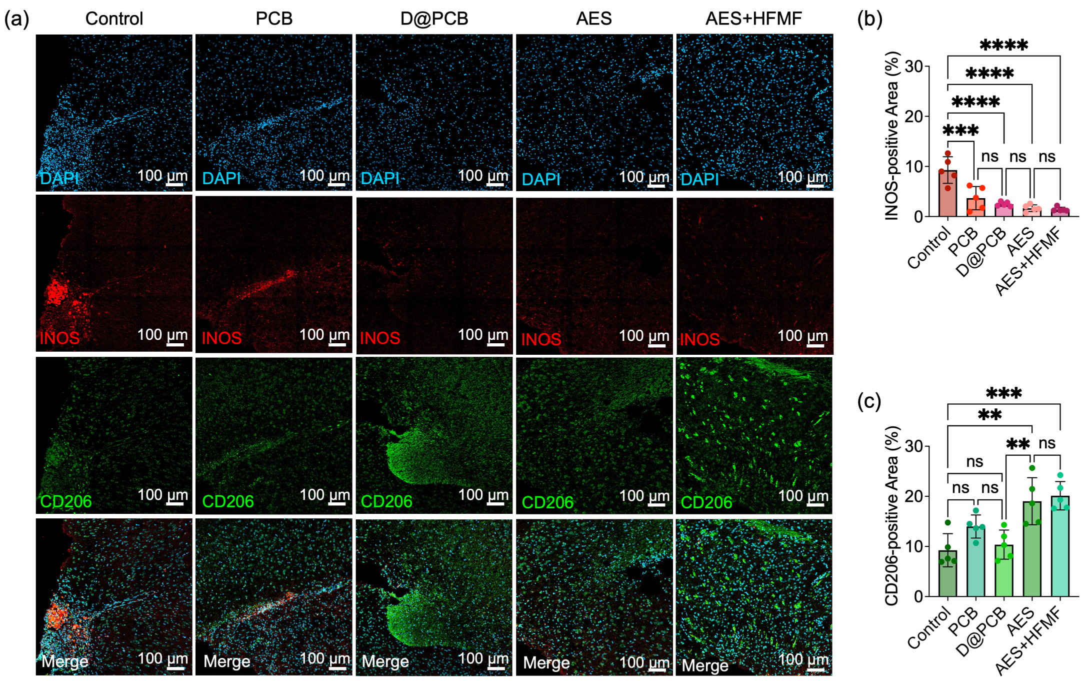

Figure S19. Long-term assessment of inflammatory polarization markers in the injured cortex at 67 days post-injury. (a) Representative immunofluorescence images showing DAPI (blue), iNOS (red), CD206 (green), and merged staining in brain sections collected 67 days after treatment. Scale bars: 100 μm. (b) Quantification of iNOS-positive inflammatory signal in the lesion-adjacent region. (c) Quantification of CD206-positive reparative signal in the lesion-adjacent region.AES+HFMF treatment was associated with reduced iNOS-positive inflammatory signals and elevated CD206-positive reparative signals relative to untreated and material-only groups, indicating a favorable shift in the chronic inflammatory microenvironment (n = 5, mean ± s.d.; one-way ANOVA with Tukey's test).

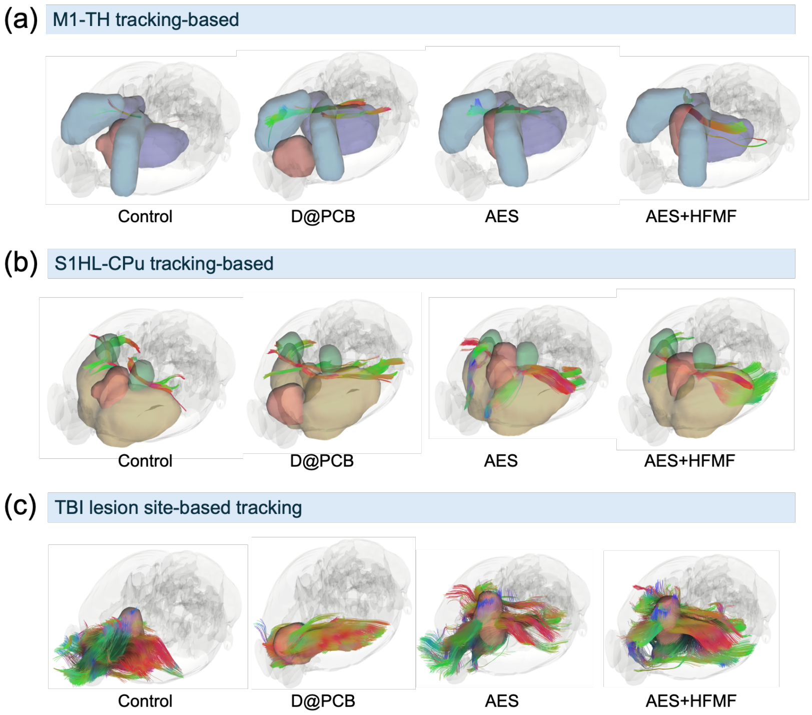

Figure S20. Representative DTI-based fiber tractography of distinct sensorimotor pathways and lesion-centered tracking in each treatment group. MRI-based diffusion tensor imaging (DTI) tractography was performed at day 14 post-TBI to visualize structural connectivity patterns across different treatment conditions. (a) M1–TH tracking-based reconstructions, highlighting corticothalamic fibers oriented along the anterior–posterior axis that mediate motor feedback between the primary motor cortex (M1, blue) and thalamus (TH, purple). (b) S1HL–CPu tracking-based reconstructions, illustrating sensorimotor fibers connecting the hindlimb somatosensory cortex (S1HL, green) to the caudate putamen (CPu, orange) along the medial–lateral axis. (c) TBI lesion site-based tracking, where seed regions were defined around the left cortical injury site to assess global fiber reorganization. Relative to the control, the TBI and untreated groups exhibited disrupted tract density and poor inter-hemispheric continuity, whereas treatment with AES and particularly AES + HFMF promoted denser, more coherent fiber projections across hemispheres and subcortical regions, indicative of partial restoration of structural connectivity.

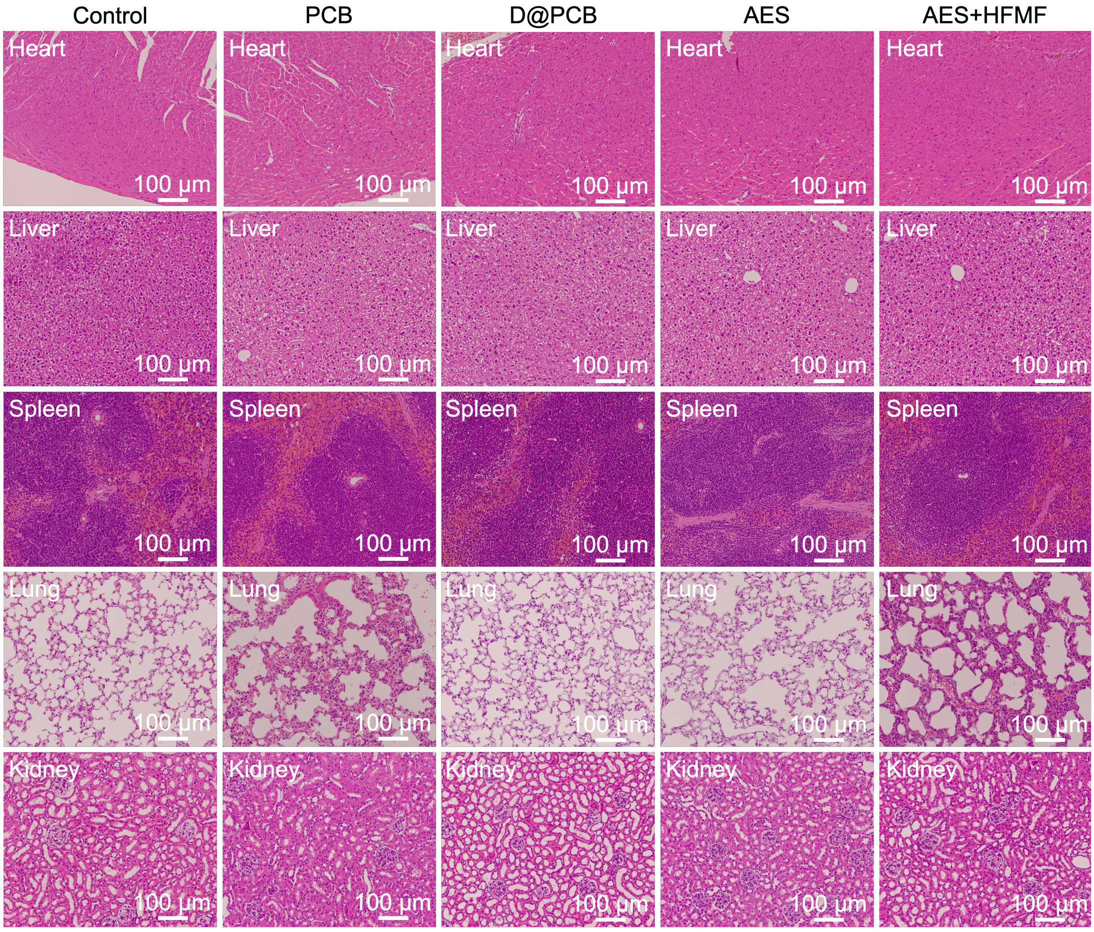

Figure S21. *In vivo* long-term systemic biosafety evaluation of the AES platform. Representative hematoxylin and eosin (H&E) stained images of major organs (heart, liver, spleen, lung, and kidney) collected from mice in different treatment groups (Control, PCB, D@PCB, AES, and AES+HFMF) at 67 days post-implantation. Microscopic examination reveals that the tissue architectures in all treated groups are well-preserved and comparable to those of the healthy control group. No observable histological abnormalities, structural damage, cellular necrosis, or inflammatory lesions were detected in any of the examined organs. These results indicate the excellent long-term biocompatibility and negligible systemic toxicity of the localized AES implantation combined with remote HFMF stimulation.
